# An agroecological structure model of compost—soil—plant interactions for sustainable organic farming

**DOI:** 10.1038/s43705-023-00233-9

**Published:** 2023-03-31

**Authors:** Hirokuni Miyamoto, Katsumi Shigeta, Wataru Suda, Yasunori Ichihashi, Naoto Nihei, Makiko Matsuura, Arisa Tsuboi, Naoki Tominaga, Masahiko Aono, Muneo Sato, Shunya Taguchi, Teruno Nakaguma, Naoko Tsuji, Chitose Ishii, Teruo Matsushita, Chie Shindo, Toshiaki Ito, Tamotsu Kato, Atsushi Kurotani, Hideaki Shima, Shigeharu Moriya, Satoshi Wada, Sankichi Horiuchi, Takashi Satoh, Kenichi Mori, Takumi Nishiuchi, Hisashi Miyamoto, Hiroaki Kodama, Masahira Hattori, Hiroshi Ohno, Jun Kikuchi, Masami Yokota Hirai

**Affiliations:** 1 Graduate School of Horticulture, Chiba University, Matsudo, Chiba 271-8501 Japan; 2 RIKEN Center for Integrative Medical Science, Yokohama, Kanagawa 230-0045 Japan; 3 Sermas Co., Ltd., Ichikawa, Chiba 272-0033 Japan; 4 Japan Eco-science (Nikkan Kagaku) Co., Ltd., Chiba, Chiba, 260-0034 Japan; 5 Takii Seed Co., Ltd., Konan, Shiga 520-3231 Japan; 6 RIKEN BioResource Research Center, Tsukuba, Ibaraki 305-0074 Japan; 7 Faculty of Food and Agricultural Sciences, Fukushima University, Fukushima, Fukushima, 960-1296 Japan; 8 RIKEN Center for Sustainable Resource Science, Yokohama, Kanagawa 230-0045 Japan; 9 Center for Frontier Medical Engineering, Chiba University, Chiba, Chiba, 263-8522 Japan; 10 Keiyo Gas Energy Solution Co., Ltd., Ichikawa, Chiba 272-0033 Japan; 11 Research Center for Agricultural Information Technology, National Agriculture and Food Research Organization, Tsukuba, Ibaraki 305-0856 Japan; 12 RIKEN, Center for Advanced Photonics, Wako, Saitama 351-0198 Japan; 13 Division of Gastroenterology and Hepatology, The Jikei University School of Medicine, Kashiwa Hospital, Kashiwa, Chiba 277-8567 Japan; 14 Division of Hematology, Kitasato University School of Allied Health Sciences, Sagamihara, Kanagawa 252-0373 Japan; 15 Division of Integrated Omics research, Bioscience Core Facility, Research Center for Experimental Modeling of Human Disease, Kanazawa University, Kanazawa, Ishikawa 920-8640 Japan; 16 Miroku Co., Ltd., Kitsuki, Oita 873-0021 Japan; 17 School of Advanced Science and Engineering, Waseda University, Tokyo, 169-8555 Japan

**Keywords:** Plant ecology, Pollution remediation, Metabolomics, Soil microbiology, Symbiosis

## Abstract

Compost is used worldwide as a soil conditioner for crops, but its functions have still been explored. Here, the omics profiles of carrots were investigated, as a root vegetable plant model, in a field amended with compost fermented with thermophilic Bacillaceae for growth and quality indices. Exposure to compost significantly increased the productivity, antioxidant activity, color, and taste of the carrot root and altered the soil bacterial composition with the levels of characteristic metabolites of the leaf, root, and soil. Based on the data, structural equation modeling (SEM) estimated that amino acids, antioxidant activity, flavonoids and/or carotenoids in plants were optimally linked by exposure to compost. The SEM of the soil estimated that the genus *Paenibacillus* and nitrogen compounds were optimally involved during exposure. These estimates did not show a contradiction between the whole genomic analysis of compost-derived *Paenibacillus* isolates and the bioactivity data, inferring the presence of a complex cascade of plant growth-promoting effects and modulation of the nitrogen cycle by the compost itself. These observations have provided information on the qualitative indicators of compost in complex soil-plant interactions and offer a new perspective for chemically independent sustainable agriculture through the efficient use of natural nitrogen.

## Introduction

Global food shortages are an urgent issue, and malnutrition remains a major cause of death in some areas. In addition, because deficiencies in trace components cause many diseases, it is necessary to develop innovative agricultural technologies to increase crop production and nutritional value [1–3]. In the meantime, it is also necessary to consider the environmental impact when promoting innovations in agricultural production. The planetary boundary framework [4] has emphasized the deterioration of biodiversity and available nitrogen and phosphorus. Traditionally, chemical fertilizers such as nitrogen and phosphorus have been considered essential and distributed in agriculture, but this approach has increased the burden on ecosystems [5, 6]. In contrast, recent studies have shown that amino acids are more critical to plants than inorganic nitrogen [5]. Considering this perspective, the importance of organic farming should be reconsidered [7, 8]. Recycling agriculture may be essential to meet the goal of sustainable development by efficiently using nitrogen and phosphorus [8, 9]. However, there is a history of contention about how the quality of organic compost affects crops in many respects [10]. Furthermore, the roles of compost in different stages of an ecosystem appear to depend on the environmental conditions [8, 11, 12]. In general, the quality of composts seems to be unstable because several different types of raw materials are fermented under uncertain fermentation conditions within composts [13–15], e.g., the contents of fermentation bacteria and moisture and other conditions of the fermentation process may vary. Therefore, although there is an assumption of the effectiveness of compost in resource recycling, it contains uncertainty due to the unstable characteristics of compost fermentation. The interaction of compost with the native soil microbiota must be considered.

Studies on thermostable and thermophilic *Bacillus* species, which may be involved in stable composting and plant health as plant growth-promoting bacteria (PGPB) candidates, have been reported [16–18]. Miyamoto, Kodama, and their joint research group have reported on thermophilic *Bacillaceae* as PGPB with antifungal activity [19] in a compost model: the model was produced at high temperature (over 70 °C as spontaneous fermentation) with marine animal resources (MAR) in a fed-batch system of bioreactors containing a thermophilic *Bacillaceae* as a stable fermentation bacterial community [19]. Interestingly, the compost had a strain that played the role of PGPB with antifungal activity [19] and reduced the accumulation of plant nitrate by denitrifying activity in soils [20]. Furthermore, oral administration of compost or its extract and the compost-derived thermophile *Bacillaceae* can improve the fecundity and quality of rodent models [21–24], livestock animals [21, 25–27], fish [28, 29], and insect models [30, 31]. Under *in vitro* anaerobic conditions, Tashiro and Sakai et al. demonstrated that compost and its derived thermophilic *Bacillus* efficiently produced optically pure _L_-lactate (100% optical purity) from nonsterilized kitchen refuse [32–35] or starch as a material [34]. These observations, including the findings of our research groups, suggest that *Bacillaceae* in compost may stably change the structure and function of symbiotic bacteria and their habitats.

**Fig. 1:**
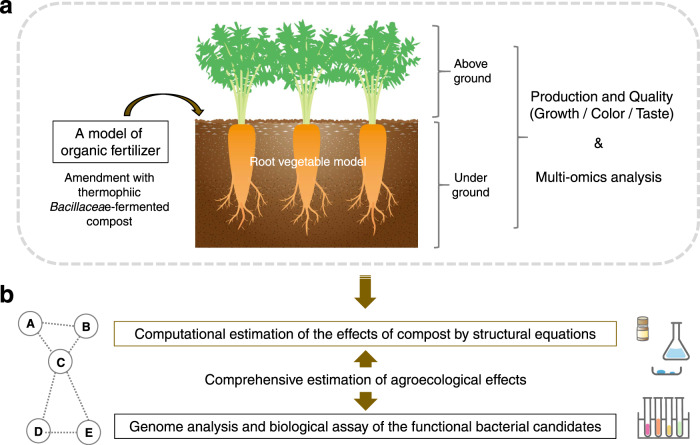
The conceptual procedures of this study. **a** Compost fermented with thermophilic *Bacillaceae* was used as a model for organic fertilizer and applied to carrots as a model for root vegetables. After the amendment, factors related to the productivity and quality of the carrots were analyzed, along with multi-omics analysis in aboveground and underground portions. **b** Based on these data, the computational structure of physiological effects by compost using structural equations was inferred. The bacterial candidates that could be involved in compost functionality based on the information were isolated. Genome analysis and biological assays on them were performed. The combination provided a comprehensive estimation of the molecular mechanisms of compost (see Fig. S1 in Supplementary information for a supplementary explanation of this legend).

Here, we aim to assess compost-soil-plant interactions using carrots. This crop model allows easy analysis of portions above and below the ground after exposure to this thermophilic *Bacillaceae* -fermented compost and to explore the active bacterial groups. Multiple omics analysis has been used as an innovative method for various research objects [5, 36]. Using integrated analyses of multiple omics datasets, including plant and soil metabolome and microbiome (Fig. 1 and S1), components linked to carrot productivity, color matrix, and taste indices are classified. The covariance structural analysis with the classified components statistically estimates the multiple regression models between plant growth indices, plant and soil metabolites, and the soil microbiota. Based on the regression model, it is inferred that *Paenibacillus* strains as nitrogen-fixing bacteria are involved in the effect of compost. After these estimates, the isolation, genome analysis, and evaluation of the biological activity of compost-derived *Paenibacillus* strains are advanced. As a result, the possibility that the calculation hypothesis was properly taken into account was anticipated. These computational procedures are used to advance biochemical experiments to effectively evaluate the complex relationships among soil, plants, and compost. The information from this study can provide an essential perspective for the construction of sustainable agricultural technologies in the future.

## Materials and methods

Two growing areas (1.8 m^2^/area) were prepared as shown in Fig. S2. The interval distance between them was separated by 30 cm. In brief, in August 2016, carrot seeds (Takii Seeds Phytorich Series, Kyo Kurenai, Takii Seeds Co., Ltd.) were sown, and seedling thinning was performed in October 2016. Thereafter, the carrots were harvested twice, the first harvest in November 2016 and the second in February 2017, and their stem and leaf weight, root weight, root diameter, root length, color, taste, the metabolites, and anti-oxidant activities were measured. Soil was collected after harvesting, and the metabolites and bacterial populations were investigated. On the basis of the omics data, correlation analyses and association analyses [37–39] were performed. Furthermore, covariance structure analysis/structural equation modeling (SEM), causal mediation analysis (CMA), and BayesLiNGAM were performed as previously described [38, 39]. Based on the predicted model, functional bacterial candidates from compost were isolated, genomic analysis was performed, and biological activity was measured. The following appropriate methods depending on the datasets were used: the Shapiro-Wilk test was used to evaluate the Gaussian distribution and to select parametric and non-parametric analyses. The F test was used to evaluate equal variances and to select the unpaired *t* test or the Welch t test as a parametric analysis. The Wilcoxon signed-rank test was used as a nonparametric analysis. Significance was declared when *P* < 0.05, and a trend was assumed at 0.05 ≤ *P* < 0.20. These calculated data were prepared using R software (version 4.0.5), Prism software (version 9.1.2), and Microsoft Office (version 16.66.1). Data are presented as the means ± SDs. Detailed testing methods not listed here are described in the Supplementary Methods.

## Results

### Harvest survey

**Fig. 2:**
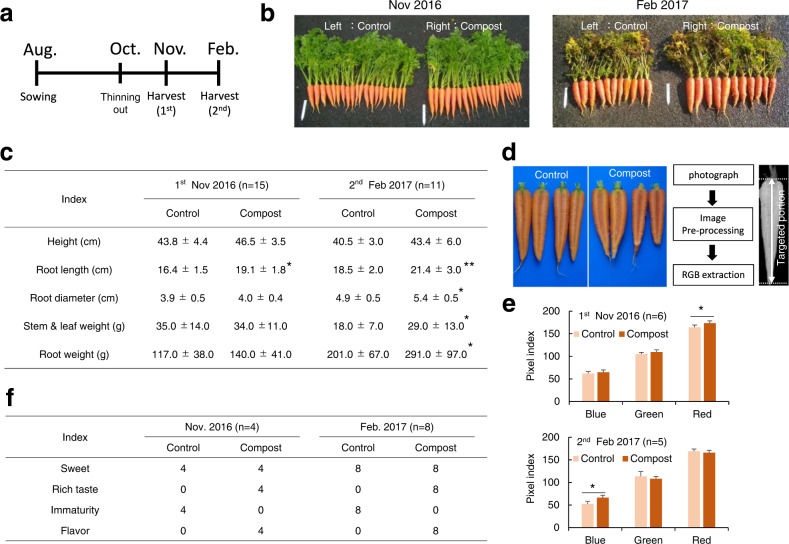
Cultivation overview. **a** Cultivation schedule. **b** Photo graph of harvested carrots. **c** Evaluated growth indices of carrots. Fresh weights are shown. **d** The procedure for color assessment. **e** Values of RGB pixel indices in the tested carrots (*n* = 6, those randomly harvested in November 2016; *n* = 5, those randomly in February 2017). **f** Results of taste evaluation. Four evaluators participated in November and eight ones in February. The asterisks are as follows: *p < 0.05; **p < 0.01.

The experiment in which the soil was fertilized with thermophile-fermented compost was planned as shown in Fig. 2a, S2a, and S2b. The growth of thinning carrots appeared to be limited by amendment with the compost one month after the start of cultivation (Fig. S2c), but then the fresh weight of the stems, leaves, and roots of carrots tended to increase (Fig. 2b). This tendency showed marked increases dependent on the duration of the exposure to the compost. A significant difference was observed in February (Fig. 2c). Interestingly, the carrots fertilized with compost tended to turn deep red. Therefore, an analysis was performed using the RGB color matrix as an index according to the procedure shown in Fig. 2d and S2. Using sample photos taken in November and February, we transformed the images as shown in Figs. S3a–d and analyzed them with the R software library ‘imager’, and found that there seemed to be differences in red, green, and blue (Fig. S3e). Therefore, we attempted to analyze the images by using the image analysis library in Python to quantify the pixel level in detail. The study of the color matrix of randomly sampled carrots showed that the rate of red coloration was significantly increased in November by compost amendment (*p* = 0.012) (Fig. 1e). The samples in February showed that the red color in the compost-amended group appeared visually to be accentuated, but the RGB color analysis revealed that the blue coloration rate increased significantly in February by the amendment (*p* = 0.031) (Fig. 2e). Furthermore, a taste survey indicated apparent differences in flavor richness and immaturity. These differences were statistically confirmed in the carrots harvested in February (X-squared=11.667, df=3, *p* = 3.441e–05) and those harvested in November(X-squared = 23.333, df = 3, *p* = 0.008617) (Fig. 2f). Regardless of the difference in RGB color, little difference in taste evaluation was observed..

These observations confirmed that the compost amendment improved the indices of carrot production and quality in this study.

### Metabolome analyses of carrot leaves and roots

**Fig. 3:**
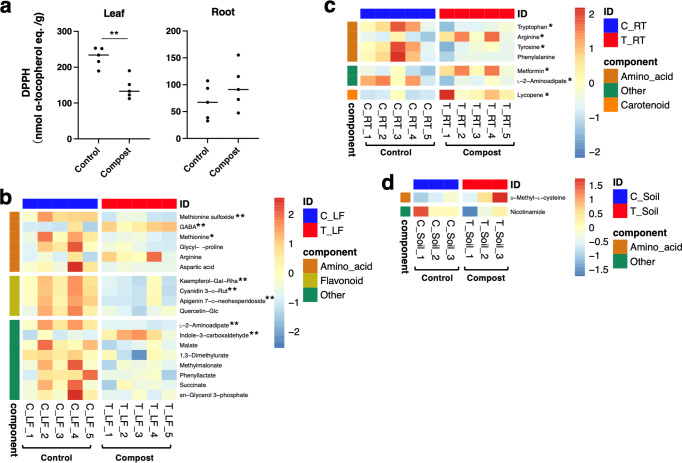
The antioxidant activity in the leaves and roots of carrots and heatmaps of correlation between the categories belonging to metabolite candidates. **a** DPPH (2,2-diphenyl-1-picrylhydrazyl) activities in the leaf and root of the carrot (*n* = 5). “Control” and “Compost” show the data under normal conditions (control group) and compost-amended conditions (compost group), respectively. **b**–**d** Metabolic differences in the leaf, root, and soil. Heatmaps show the relative abundances of the metabolites in each cluster: the control (blue) and test groups (compost-amended group) (red). The representative metabolites in the leaf and the root in each group are shown (comparison of the categories belonging to metabolites with *p* < 0.1 as significant values). The metabolites in the soil are shown (comparison of the categories belonging to metabolites with *p* < 0.2 as significant values). Significant values were shown as follows: *p < 0.05; and **p < 0.01.

Based on these results, a detailed nutritional analysis was conducted on the February carrot samples. Because carotenoids and polyphenols generally alter the color of carrots [40, 41], these concentrations were examined. The results confirmed that the concentrations of α-carotene (*p* = 0.0103 vs. control) and lycopene (*p* = 0.0146 vs. control) significantly increased. (Fig. S4). Beta-carotene tended to increase, although not significantly (*p* = 0.1188 vs. control). The application of thermophile-fermented compost appeared to increase the 2,2-diphenyl-1-picrylhydrazyl (DPPH) content by 40% (*p* = 0.2612) in the roots, although not significantly. At the same time, it significantly decreased the DPPH content by 40% in the leaves (*p* = 0.0014) (Fig. 3a). On the basis of these evaluations, metabolome analyses of carrot leaves and roots were performed. Additionally, correlation analysis was performed on the basis of all analyzed data. As a result, the correlations of amino acids, flavonoids, and phenylpropanoids were different between the control and compost groups (Fig. S5).

In detail, the levels of metabolic compound candidates changed as follows: the levels of 4-amino-butyric acid (GABA), as an amino acid-related metabolite, increased significantly in the leaf after compost exposure (*p* = 0.0072), but methionine sulfoxide and methionine levels decreased considerably (*p* = 0.0007). The levels of the metabolites annotated as kaempferol-Gal-Rha, cyanidin 3-O-Rut, apigenin 7-O-neohespseridoside, and quercetin-Glc as flavonoids in the leaf were significantly decreased by exposure (*p* = 0.0023; *p* = 0.0031; *p* = 0.0035; and *p* = 0.0147, respectively) (Fig. 3b). For other categories, indole 3-carboxyaldehyde increased considerably by exposure (p = 0.009), but l-2-aminoadipate, malate, 1,3-dimethylurate, methylmalonate, and phenyllactate decreased (*p* = 0.0079; *p* = 0.0106; *p* = 0.0230; *p* = 0.0410; and *p* = 0.0461, respectively). In roots (Fig. 3c), tryptophan, phenylalanine, tyrosine, and l-2-aminoadipate levels, as amino acids and nitrogen metabolites, were significantly reduced by exposure (*p* = 0.0177; *p* = 0.0952; *p* = 0.0449; and *p* = 0.0355, respectively), and arginine levels were significantly increased (*p* = 0.0315). The correlation between these individual metabolites was also clearly different (Fig. S6).

Thus, such an increase or decrease in common metabolites was not necessarily confirmed. However, at least the compost amendments significantly changed leaf and root metabolites, especially leaf flavonoids involved in antioxidant activity. It has become possible to classify characteristic metabolites in carrot leaves and roots depending on the cultivation conditions.

### Soil omics analysis

To evaluate the relationships among leaves, roots, and soils, soil omics analyses were also performed. Due to the difference in the target and analysis conditions, metabolome analysis was performed using a device different from the one used for the leaf and root. Of the amino acids and related compounds, _*S*_-methyl- _L_-cysteine had an increased tendency in the composting group, and the levels of nicotinamide tended to decrease (Fig. 3d).

**Fig. 4:**
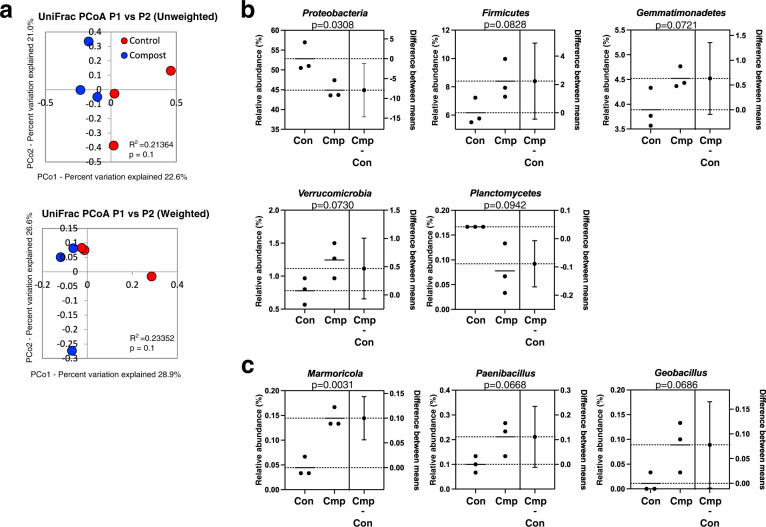
Soil bacterial populations. **a** UniFrac graph (unweighted and weighted) showing β-diversity in the control and test groups. The estimation plot of the bacterial population in the soil of the control and test groups. Relative abundances of the (**b**) phyla and (**c**) genera (*p* < 0.1; >0.1% as the maximum bacterial population).

Subsequent analysis of the soil bacterial population suggested that the bacterial diversity differed between conditions with and without the application of compost (Fig. 4a and S7a). There was little change in α-diversity (Fig. S7a). However, the beta diversity tended to change, although not significantly (Fig. 4a). Some changes in the bacterial populations were observed that were not always significant differences (Fig. 4 and S8). Evaluation at the phylum level showed that the phylum *Proteobacteria* as a dominant bacterial group decreased significantly after compost amendment. The levels of the phyla *Gemmatimonadetes*, *Verrucomicrobia*, *Firmicutes, Elusimicrobia*, and *Thaumarchaeota* tended to be high (Fig. 4b and S8a). In contrast, the phyla *Planctomycetes*, *Acidobacteria*, *Spirochetes*, and *Deinococcus-Thermus* tended to decrease. At the genus level (Fig. 4c and S8b), the abundance of the genus *Marmoricola* increased significantly. Furthermore, the genera *Paenibacillus*, *Geobacillus*, *Panacagrimonas*, *Nocardiodes*, and *Phycicoccus* tended to increase.

Therefore, these observations suggested that the amendment of the compost caused changes in the bacterial populations of the soil and altered the metabolism of the soil.

### Association analyses and covariance structure analyses

An association analysis was performed based on production indices, color matrix, and omics data, and factors strongly related to compost administration were selected (Fig. S9). In terms of appearance and taste, compost fertilization increased leaf SPAD and color, while in terms of taste, rich taste and flavor increased and immaturity decreased (Fig. S9a). In leaves, 2-aminoadipate, 3-hydroxyanthranilate, threo-3-methylaspartate, methionine sulfoxide, and DPPH activity decreased, while in roots, DPPH activity andcarotenoid increased and guanine decreased (Fig. S9b). In terms of the relationship between soil metabolites and flora, factors with high lift values were identified at the phylum and genus levels, and at the phylum level, it was calculated that compost amendment tended to increase phyla *Firmicutes*, *Gemmatimonadetes*, *Planctomycetes*, *Proteobacteria*, *Thaumarchaeota*, and *Verrucomicrobia*. In addition, the genera *Geobacillus*, *Legionella*, *Marmoricola*, *Paenibacillus*, *Phycicoccus*, and *Ramlibacter* were calculated to be increased by the amendment. Based on the classified data together with the results obtained in Fig. 3, S5, and S6, structural equation analysis was carried out to understand the basic structure of the skeleton in this complex system.

**Fig. 5:**
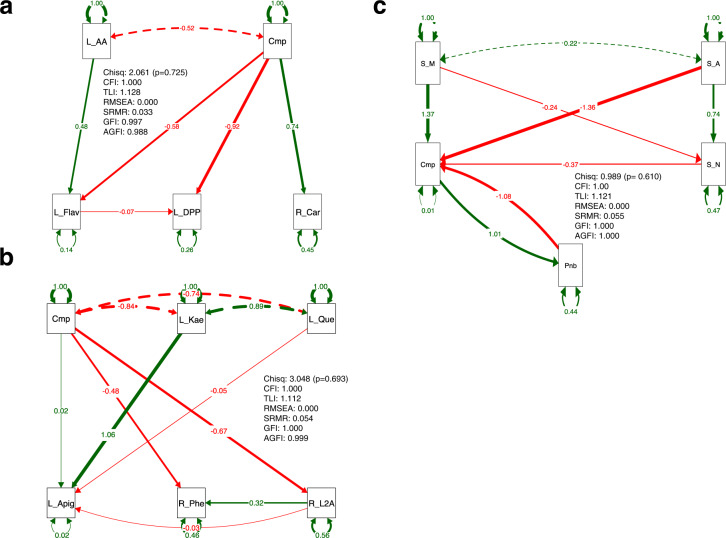
The relationship of the leaf and root metabolite candidates associated with compost amendment was visualized by structural equation modeling in the regression groups selected in Tables S1 and S2. Standardized β coefficients are reported. The green and red colors show positive and negative associations, respectively. **a** Shows the sempath linked with amino acids, carotenoids, flavonoids, DPPH activity, and compost. **b** Shows the sempath linked with apigenin 7-O-neohesperidoside, kaempferol-Gal-Rha, and quercetin Glc as flavonoids in the leaf and L-2-aminoadipate and phenylalanine in the root and compost. **c** Shows the sempah linked with soil metabolites, *Paenibacillus*, and compost.The abbreviations are as follows: Cmp, compost; L_AA, total amino acid contents in the leaf; L_Fav, total flavonoid contents in the leaf; L_DPP, DPPH activities in the leaf; R_Car, total carotenoid contents in the root; L_Apig, apigenin 7-O-neohesperidoside in the leaf; L_Kae, kaempferol-Gal-Rha in the leaf; L_Que, quercetin Glc in the leaf; R_L2A, L-2-aminoadipate in the root; R_Phe, phenylalanine in the root; Pnb, *Paenibacillus*; S_A, L-2 aminoadipate; S_N, nicotinamide; S_M, *S*-methyl L-cysteine. The fit indices are shown in the path as follows: Chiq, chi-square χ^2^; *p* value, *p* values (chi-square); CFI comparative fit index, TLI Tucker–Lewis index, RMSEA root mean square error of approximation, SRMR standardized root mean residual, GFI goodness-of-fit index, AGFI adjusted goodness-of-fit index.

The structural equation calculates the combination showing the optimum value. Among the calculated models for DPPH as an indicator of antioxidant activity, the multiple regression model with amino acids, flavonoids, and carotenoids (Fig. 5a and S10a) had the highest optimal fit index values (Table S1). Therefore, the structural equation for flavonoids was calculated with phenylalanine, a flavonoid precursor, focusing on the factors that actually changed in the above observations. As a result, apigenin 7-O-neohesperidoside, kaempferol-Gal-Rha, and quercetin_Glc, as leaf flavonoids, root l-2-aminoadipate, and phenylalanine formulated the optimal structural equation (Fig. 5b and S10b) rather than the other inferior models (Table S2). These estimations suggested that compost amendments are structurally involved as a group in the concentration of flavonoids, amino acids, and carotenoids in carrots, as well as in the antioxidant activity of carrots.

Next, the relationships between the changes observed in the compost-treated soil were constructed by modeling structural equations. The soil bacterial candidates and soil metabolites that showed representative changes by compost amendment were selected in the above experiment. The relationship of the soil factors associated with compost amendments was visualized as optimal SEM (Fig. 5c and S10c): an optimal model that included the genus *Paenibacillus*, dl-2-aminoadipate, nicotinamide, and _*S*_-methyl _L_-cysteine showed good fit indices compared to those of the other models (Table S3). These selected components within the optimal models were not necessarily composed of all statistically significant ones (Fig. 4 and S11).

In examining the degree of optimization of these as a group, calculations using two types of statistical causal inference, causal mediation analysis (CMA) and BayesLiNGAM, were performed to test whether the factor groups on the structural equations were important as a group. CMA revealed that a single mediation (indirect) effect (ACME) was not calculable or without significant values (Tables S4, S5, and S6). These calculated results estimated a strong relationship for each group. Therefore, the causal interactive relationships within each optimal model of the leaf, root, and soil were investigated based on a calculation by BayesLiNGAM (Fig. S12). The highest causal interactive relationships (top six) were mainly shown from compost but not always. This may mean that the compost supports it while the physiological responses of the carrot and/or the soil themselves are prioritized. These results pointed to the importance of the optimal model as a whole group, as well as the optimal model group estimated for the leaf and root.

Therefore, nitrogen compounds were involved in these structural equations for leaves, roots, and soil. Based on these observations, the genus *Paenibacillus* derived from compost was explored, followed by a search for its function within the compost itself.

### Genome profiles associated with plant growth promotion

**Fig. 6:**
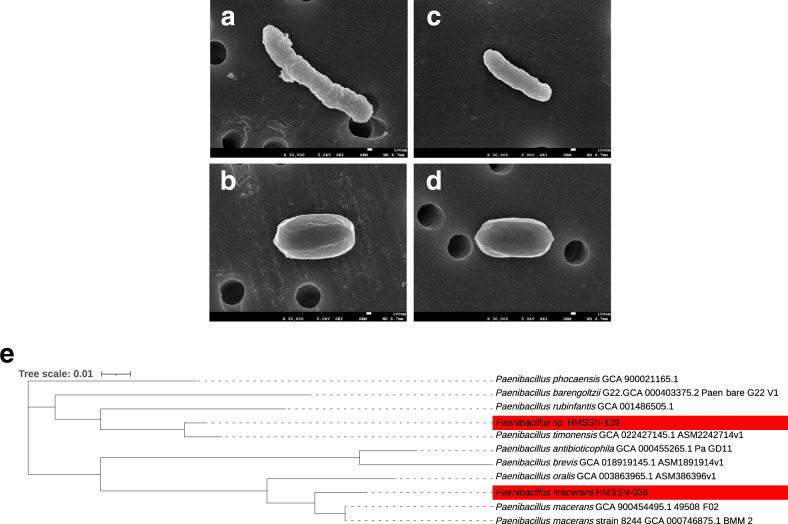
Electron micrographs and phylogenetic relationships of isolated *Paenibacillus* strains in this study. **a** Scanning electron microscopy image of vegetative type *Paenibacillus macerans* HMSSN-036 stain. **b** Scanning electron microscopy image of spore type *Paenibacillus macerans* HMSSN-036 stain. **c** Scanning electron microscopy image of vegetative type *Paenibacillus* sp. HMSSN-139 stain. **d** Scanning electron microscopy image of spore type *Paenibacillus* sp. HMSSN-139 strain. **e** Whole-genome phylogenetic analysis of isolated *Paenibacillus* strains was performed. A phylogenetic tree was constructed by the neighbor-joining method. The isolated *Paenibacillus* strains are marked in red.

As described above, the SEM results suggested that the genus *Paenibacillu*s may be a necessary component of compost for the promotion of plant growth. Therefore, we isolated compost-derived colonies that tested positive for a nitrogen fixation gene and were able to select two strains that had a nitrogen fixation reaction from compost-cultured colonies. The compost-derived strains *Paenibacillus macerans* HMSSN-036 and *Paenibacillus* sp. HMSSN-139 were identified, and their genomes were analyzed: the former, a strain closely related to *Paenibacillus macerans* (identity 99.0%); the latter, a candidate as a new species belonging to *Paenibacillus*. In Fig. 6a–d, electron micrographs of these strains showed the spore- and vegetative-forms. The molecular phylogenetic tree of these isolates is shown in Fig. 6e. Phylogenetic analysis with mash distance (Fig. S13) showed that the isolated *Paenibacillus* strains were classified into weak sequences for *P. curdianolyticus* and *P. kobensis* detected by meta-sequence analysis of the 16 S bacterial rRNA gene sequence. Their population was not detected in the control and was 0.022 ± 0.011% in the compost group (*p* = 0.116). These observations indicated that the isolated *Paenibacillus* strains did not necessarily coincide with the sequence of *Paenibacillus* species increasing in the compost-amended soil. Still, it is interesting that the same *Paenibacillus* increased in the soil. Furthermore, genome analyses showed the presence of genes for nitrogen fixation, auxin production, phosphate solubilization, and siderophore reactions, such as PGPR (Table 1 and S7–S10). Based on genomic information, biological assays were performed for auxin production, siderophore reaction, and phosphate solubilization and all of these assays were demonstrated to be positive (Fig. S14). Therefore, the genes detected in isolated *Paenibacillus* strains could be functional for nitrogen fixation, auxin production, siderophore reaction, and phosphate solubilization in the bacterium.

**Table 1:** Functional genes identified based on the genomic data of *Paenibacillus* sp. isolated from the thermophile-fermented compost (see Figs. S13 and S14 and Tables S7–S10 in the supplementary information).

Function	Isolated strain names	
	*Paenibacillus macerans* HMSSN-036	*Paenibacillus* sp. HMSSN-139
Nitrogen fixation	nitrogen fixation protein NifB	nitrogen fixation protein NifB
	nitrogenase molybdenum-cofactor synthesis protein NifE	nitrogenase molybdenum-cofactor synthesis protein NifE
	nitrogenase iron protein NifH	nitrogenase iron protein NifH
	nitrogenase molybdenum-iron protein NifN	nitrogenase molybdenum-iron protein NifN
	nitrogen fixation protein NifU	nitrogen fixation protein NifU and related proteins
	nitrogen fixation protein NifX	nitrogen fixation protein NifX
Nitrogen cycle cofactor	nitrite transporter NirC	nitrite transporter NirC
Auxin production	auxin efflux carrier	auxin efflux carrier
	indole-3-glycerol phosphate synthase	indole-3-glycerol phosphate synthase
	auxin-induced protein	-
Phosphate solubilization	inorganic phosphate transporter, PiT family	inorganic phosphate transporter, PiT family
Siderophore reaction	Fur family transcriptional regulator, ferric uptake regulator	Fur family transcriptional regulator, ferric uptake regulator

These results suggest that compost-derived *Paenibacillus* strains may exhibit PGPR effects.

### Evaluation of the effects of compost itself on the promotion of plant growth

The nitrogen cycle function of compost with nitrogen-fixing *Paenibacillus* was then evaluated with in vitro soil models. Two functional evaluations of nitrogen fixation and reduction of nitrous oxide generation were conducted.

First, as shown in Fig. S15a, the nitrogen fixation capacity was evaluated in soil with the stable nitrogen isotope ^15^N added. The *Arabidopsis thaliana* model plant was cultured to assess the effects on nitrogen fixation, and a growth promotion effect, although not significant, was observed (Fig. S15b). Under such conditions, nitrogen fixation rates of approximately 20% were possible in the plant and soil. Based on these observations, it is possible that the soil modulates nitrogen fixation in the field after exposure to compost. The results of the elemental analysis of the soil immediately after the cultivation of the carrot-grown soil in this field test changed significantly, although it was tested after it was well mixed with the soil in the control and test areas prior. Total nitrogen tended to increase slightly, and the EC value related to the nitrate concentration was higher in the test area (Table S11). It should be noted that the two groups of soils were well mixed by a skilled person prior to the cultivation, although no analysis of the initial soil was conducted.

Next, a system was created to measure the amount of N_2_O generated from the soil (Fig. S15c). Fungi having cytochrome P450nor that generates N_2_O gas are considered as a major biological factor in the generation of greenhouse gases [42–44]. Potato dextrose power, which can generally be used as a medium for fungi, was added to the soil for the test. The test results confirmed that the conditions with added compost showed markedly reduced N_2_O generation compared to that of the control group (soil only with fungi and PDA) (Fig. S15d). Reproducibility tests were conducted in three different soil containers. The ratio of fungi present in the prepared soil was investigated. The results of the NGS analysis of the prepared soil indicated that it mainly contained *Gibberella* sp. (66.1%) and *Fusarium* sp. (28.7%) as fungi. Since N_2_O content was approximately the same as those under the condition with no nutritional source (PDA) of fungi as a reference test, it was suggested that the inhibitory effect was even greater. These results are based on the test laboratory level. In contrast, measuring nitrous oxide at the field level and exploring the mechanism of action of complex microorganisms is costly and time-consuming. At any point, the phenomena identified in this study are of social significance. In contrast, the use of such a laboratory system suggests that it is possible to assess the potential for N_2_O generation in soil collected from the field in the laboratory without measuring the volume of N_2_O at the field level.

In addition to these results, the growth of carrots in this field experiment was consistent with the fact that the isolated *Paenibacillus* strains from the compost were positive for producing the plant hormone auxin, phosphate solubilization and siderophore reactions (Fig. S14a). The relationship between the number of bacteria and auxin production was also verified at the *in vitro* level (Figs. S14b and S14c). In addition, it should be noted that the soil tested immediately after harvesting the carrot fields confirmed an increase in PAC and a decrease in iron concentration (Table S11). The field test results were consistent with the compost-derived *Paenibacillus* strains being positive for phosphate solubilization (Fig. S14d) and siderophore reactions (Fig. S14e).

Thus, the function of the compost was verified in the field test and had similarities with the results of the *in vitro* test as complementary data.

## Discussion

Here, we succeeded in deriving structural equations related to nitrogen metabolism in leaves, roots, and soil as the effect of thermophile-fermented compost. In the structural equation, it was assumed that *Paenibacillus*, a candidate for nitrogen fixation, might be involved, and we succeeded in isolating *Paenibacillus* strains derived from the compost. Furthermore, based on genomic analysis and evaluation of biological activity, the possibility of nitrogen circulation in plants and soil by the compost used here was confirmed.

In plants, carotenoids, flavonoids, and amino acids are the constituents of leaf and root metabolites in the structural equation. Carotenoids and polyphenols (containing flavonoids) are known to affect the color of carrot, as well as nutrients [40, 41]. In general, previous studies reported that carrot coloration is often explained by genetic variation influencing qualitative changes in carotenoids and polyphenols [45, 46]. However, it was found that compost fertilization can affect the color of carrots of the same variety. Although the mechanism of action is not known, it is likely that differences in metabolites, specifically carotenoids, flavonoids, and amino acid composition, may be responsible. In particular, the structural equations of amino acids and flavonoids were inferred. The optimal compost-linked SEM linked with apigenin 7-O-neohesperidoside, kaempferol-Gal-Rha, and quercetin Glc as leaf flavonoids was consistent with that phenylalanine being the precursor of flavonoids [47, 48]. The model provided a new insight into using l-2-aminoadipate as a nitrogen compound. 2-Aminoadipate candidates commonly detected in leaves, roots, and soil were linked to the estimated SEM candidates (Tables S2 and S3). l-2-aminoadipate was significantly altered in both leaves and roots with the addition of compost, but dl-2-aminoadipate was not always changed in the soil. Furthermore, the difference in the l and dl types may depend on different equipment, and the cause was unclear. In any case, the estimated SEM candidates with 2-aminoadipate had relatively suitable fit index values. Since 2-aminoadipate is associated with the activity of lysine–oxoglutarate reductase [49], the relationship with lysine was reassessed. 2-Aminoadipate was not only reduced in leaves and roots (Fig. S16a), but the proportion in leaves and roots increased conversely in the roots (Fig. S16b). Lysine tended to increase in roots (Fig. S16c), and the proportions in leaves and roots also tended to increase in roots (Fig. S16d). Root lysine was not adopted as a factor in the structural equation in Fig. 5b because it was not significant (*p* < 0.05, Fig. 3c) and did not have a high lift value in the association analysis (Fig. S9). However, the structural equation, including lysine, was calculated on the basis of the information on physiological function. As shown in the results (Table S12), the model that included some regression model formulas showed the optimum value, along with the models with a metabolite annotated as metformin and arginine, rather than the model that contained lysine and aminoadipate. In particular, metformin and arginine also increased in the roots (Fig. 3c). Metformin is a component of therapeutic agents for the treatment of diabetes [50] and an anti-aging therapy candidate [51]. Previous reports of plant-derived metformin are notable [52, 53]. Arginine is potentially therapeutic for cardiovascular disorders [54]. These functional titers need to be recognized with more care that considers their physiological efficacy. However, it may make sense that nitrogen metabolites have been detected after compost amendment with nitrogen-fixing *Paenibacillus*.

In the structural equation of leaves and roots, carotenoids appeared to be directly affected by compost, but the relationship between other metabolites was not statistically clarified. Due to the complex metabolic relationship of the whole plant, it may be fitted according to the structural equation. At least, the soil sampled immediately after carrot cultivation showed reduced iron (Table S11), and compost-derived *Paenibacillus* may be involved in exhibiting a siderophore response to promote iron absorption. In addition, it was reported that the biosynthesis of carotenoids was controlled by cytochrome P450 [55], which could have heme with iron as an essential component [56].

In the structural equations between soil metabolites, soil bacteria, and compost, the decreasing trend of nicotinamide and the increasing trend of _S_-methyl _L_-cysteine deserve attention. Nicotinamide has been reported to be involved in plant growth [57–59], biological defense [60], and antioxidant activity [61]. _S_-methyl _L_-cysteine sulfoxide, a derivative of s-methyl l-cysteine, is an attractant for plant parasitic nematodes [62], which reduce plant growth and productivity. However, s-methyl l-cysteine sulfoxide, a precursor of the soil fumigant dimethyldisulide (DMDS) [63], is produced by plants [64] and bacteria [65]. Although DMDS is a soil fumigant that controls soil-borne pathogens and nematodes, it has been pointed out that it may not function depending on the microbial environment of the soil [63]. Therefore, investigating the significance of these metabolites is an interesting perspective. In addition, some *Paenibacillus* strains are known to have a defensive effect on nematodes [66, 67]. In fact, the thermophile-fermented compost used in this experiment was less likely to cause nematode damage in another field test and made it difficult to form root knots as a detriment (Fig. S17). These results appeared to be different from those involving nematodes already reported [68], although under the other experimental conditions. Nevertheless, the commonalities here may confirm a novel aspect of the defense against root-knot nematode damage using the *Paenibacillus*-harboring compost in the structural equation.

As previously reported, the 16 S rRNA sequence of the genus *Paenibacillus* was one of the bacterial genera once it was found in compost [19]. In this study, two strains of *Paenibacillus* spp. from compost were newly isolated as nitrogen-fixing bacteria. Genome data were consistent with the structural equations and production results in this experiment, although not all. Except for the genes for nitrogen fixation, auxin production, phosphate solubilization, siderophore reaction, and related function, genes related to GABA and/or isopropylmalate production (Table S9) were notable. GABA plays a crucial role in plant drought tolerance [69] and pathogen and insect attacks [70] and has been suggested to play a vital role in nitrogen fixation in seagrass [71], even though seagrass is not a terrestrial plant. 2-Isopropylmalate synthase (IPMS) is involved in the synthesis of 2-isopropylmalate [72]. Notably, IPMS is involved in the biosynthesis of the amino acid leucine [73] and flavor compounds in apple. Although the plant species are different, they do not contradict the results in this study. In any case, although GABA was detected in plants, the abundance of GABA in the soil was extremely low. Furthermore, the abundance of 2-isopropylmalate in the soil was also extremely low, resulting in difficult discussions based on concentration in the soil.

The identical relationship between the genus *Paenibacillus* detected in soil omics analysis (Fig. 4c) and isolated *Paenibacillus* strains derived from compost (Table 1) should be confirmed by mash distance analysis (Fig. S13). Unfortunately, it was impossible to verify that the isolated *Paenibacillus* strains from the compost were consistent with the sequences detected in the carrot soil. However, it was interesting that the abundance of bacteria belonging to the genus *Paenibacillus* found in compost also increased in compost-treated soil. Quorum sensing is commonly defined as a signaling network between bacteria that regulates their function as a bacterial group depending on the density of a given species [74], and notably, known species belonging to *Paenibacillus* [75] and *Bacillus* [76] carry quorum sensing genes. Thus, it may be suggested that this mechanism might contribute to plant growth and that closely related *Paenibacillus* species detected in the soil from the carrot experiment play a role in the whole group of genera.

This study evaluated the impact of *Paenibacillus*-harboring compost on the nitrogen cycle in the soil, which cannot be carried out in field trials. As a result, nitrogen fixation capacity and reduction in N_2_O were confirmed. In recent years, it has been suggested that *Paenibacillus polymyxa* may be involved in the suppression of N_2_O generation [77], but the results of this study confirmed the effect of the compost itself. The N_2_O in the soil is generated by cytochrome P450 derived from fungi [42, 43], which are often pathogens of soil origin. Notably, a bacterium producing cyclic lipopeptides that suppress growth in fungi [19] was also present in the compost used in this experiment. Thus, thermophile-fermented compost is expected to be a potential candidate for ecological biostimulants. Following this research, it should be necessary to explore the conditions under which compost can be used more efficiently.

**Fig. 7:**
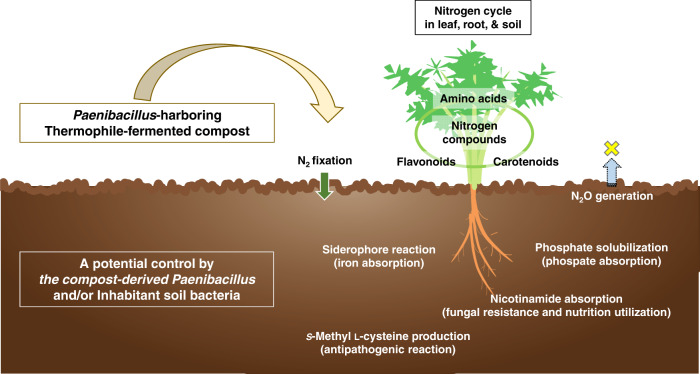
A putative model consequential in this study. The interaction of compost-derived *Paenibacillus*, soil symbionts and their metabolites is visualized.

Fig 7 shows an inference model obtained from the results of this survey. This result shows that the introduction of compost along with *Paenibacillus* affects the nitrogen cycle of the soil and the plant body inherent in the soil. Expressly, the model assumes that nitrogen fixation and associated suppression of nitrous oxide production occur in the soil and that the absorption of phosphate, iron, and nicotinamide, which are plant nutrient sources, is enhanced. As a result, increases or decreases in amino acids, flavonoids, and carotenoids involved in the nitrogen cycle would be expected to be affected. In addition, it has recently been suggested that nicotinamide may confer disease resistance to pathogenic fungi [78]. Furthermore, an environmentally friendly mechanism is assumed by increasing the content of _*S*_-methyl _L_-cysteine, which has antipathogenic properties. Taking these points into account, it was again noteworthy that a complex functional cascade of compost is computationally estimated by the structural equations, starting from *Paenibacillus*, together with the functions of its possessed genes, namely nitrogen fixation, phosphate solubilization, and iron utilization (siderophore reaction).

There is a global need for sustainable organic farming. In this study, compost fermented at high temperatures was used as a model organic fertilizer. Integrated omics analyses were conducted using carrots as a crop model, allowing easy analysis of the portions above and below the ground. Based on the characteristics of omics data, SEM estimated the optimal models that the compost itself and/or the influenced rhizosphere improve the productivity and quality of the crop and environmental conditions, such as nitrogen fixation and denitrification with reduced greenhouse gases. These statistical inferences provide a novel perspective on the potential use of sustainable compost.

Interestingly, recent reports have estimated that this compost may also be effective in the flourishing of seagrasses by machine learning and causal inference targeting the symbiotic bacteria of seagrass sediments [39]. The similarities to this study are not clear, but there may be an overarching role of compost to flourish broad plants beyond the categories of terrestrial plants and seagrasses.

## Supplementary information

Supplementary information

Supplementary Methods

## Data Availability

Raw files of the bacterial V1–V2 16 S rRNA data are deposited in the DNA Data Bank of Japan (DDBJ) under NCBI Bio-Project accession numbers PRJDB11391 (PSUB014602) (BioSample accession numbers: SAMD00291237-SAMD00291242). Raw files of the bacterial genome data are also deposited in DDBJ under NCBI Bio-Project accession number PRJDB11391 (PSUB014602) (BioSample accession numbers: SAMD00422992-SAMD00422993). Raw files of the fungal ITS data are deposited in DDBJ under the NCBI Bio-Project accession number PRJDB13243 (PSUB01706) (BioSample accession numbers: SAMD00448670-SAMD00448671).
