# Supplementary material for: An agroecological structure model of compost—soil—plant interactions for sustainable organic farming
Source: ISME Commun. 2023 Mar 31;3:28. doi: 10.1038/s43705-023-00233-9 (PMC10066230; doi:10.1038/s43705-023-00233-9)
Supplement: Supplementary file 1 — Supplementary information [file 43705_2023_233_MOESM1_ESM.pdf]

Supplementary Information

An agroecological structure model of compost-soil-plant interactions for sustainable organic farming

Miyamoto*[1,2,3,4], Katsumi Shigeta[5], Wataru Suda[2], Yasunori Ichihashi[6], Naoto Nihei[7], Makiko Matsuura[1,3], Arisa Tsuboi[4], Naoki Tominaga[5], Masahiko Aono[5], Muneo Sato[8], Shunya Taguchi[9], Teruno Nakaguma[1,3,4], Naoko Tsuji[3], Chitose Ishii[2,3], Teruo Matsushita[3,4], Chie Shindo[2], Toshiaki Ito[10], Tamotsu Kato[2], Atsushi Kurotani[8,11], Hideaki Shima[8], Shigeharu Moriya[12], Satoshi Wada[12], Sankichi Horiuchi[13], Takashi Satoh[14], Kenichi Mori[1,3,4], Takumi Nishiuchi[15], Hisashi Miyamoto[3,16], Hiroaki Kodama[1], Masahira Hattori[2,17], Hiroshi Ohno[2], Jun Kikuchi*[8], Masami Yokota Hirai*[8]

Affiliations:

1. Graduate School of Horticulture, Chiba University, Matsudo, Chiba 271-8501, Japan

2. RIKEN Center for Integrative Medical Science, Yokohama, Kanagawa 230-0045, Japan

3. Sermas Co., Ltd., Ichikawa, Chiba 272-0033, Japan

4. Japan Eco-science (Nikkan Kagaku) Co., Ltd., Chiba, Chiba 260-0034, Japan

5. Takii Seed Co., Ltd., Konan, Shiga 520-3231, Japan

6. RIKEN BioResource Research Center, Tsukuba, Ibaraki 305-0074, Japan

7. Faculty of Food and Agricultural Sciences, Fukushima University, Fukushima, Fukushima 960-1296, Japan

8. RIKEN Center for Sustainable Resource Science, Yokohama, Kanagawa 230-0045, Japan

9. Center for Frontier Medical Engineering, Chiba University, Chiba, Chiba 263-8522, Japan

10. Keiyo Gas Energy Solution Co., Ltd., Ichikawa, Chiba 272-0033, Japan

11.Research Center for Agricultural Information Technology, National Agriculture and Food Research Organization, Tsukuba, Ibaraki, Japan, 305-0856

12. RIKEN, Center for Advanced Photonics, Wako, Saitama, Japan, 351-0198

13. Division of Gastroenterology and Hepatology, The Jikei University School of Medicine, Kashiwa Hospital, Chiba, Japan.

14. Division of Hematology, Kitasato University School of Allied Health Sciences, Sagamihara, Kanagawa 252-0329, Japan

15. Division of Integrated Omics research, Bioscience Core Facility, Research Center for Experimental Modeling of Human Disease, Kanazawa University, Kanazawa, Ishikawa, 920-8640, Japan

16. Miroku Co., Ltd., Kitsuki, Oita 873-0021, Japan

17. School of Advanced Science and Engineering, Waseda University, Tokyo169-8555, Japan

* Cocorrespondence:

Hirokuni Miyamoto Ph.D., hirokuni.miyamoto@riken.jp; h-miyamoto@faculty.chiba-u.jp

Jun Kikuchi Ph.D., jun.kikuchi@riken.jp

Masami Yokota Hirai Ph.D., masami.hirai@riken.jp

Figure contents

Fig. S1 Experimental scheme in this study.

Fig. S2 Overview of Growing conditions.

Fig. S3 Pre-processing for image analysis.

Fig. S4 The contents of primary carotenoids in the roots of carrots.

Fig. S5 Heatmaps of correlations between the amino acid, carotenoid, flavonoid, DPPH activity in the leaves and roots.

Fig. S6 Heatmaps of correlations between metabolites in the leaves and roots shown in Fig. 3.

Fig. S7 Bacterial diversity in the soil after cultivation of carrot.

Fig. S8 The population of phyla and genera in the soil of the control and compost groups.

Fig. S9 Association networks in the leaves and root metabolites, and the soil bacteria by compost administration.

Fig. S10 The path of Fig. 5 calculated by the function "sem".

Fig. S11 The relative area of metabolites used for optimal structural equations.

Fig. S12 The top six causal structural groups for SEM (Fig. 5) estimated by BayesLiNGAM.

Fig. S13 Phylogenetic relationship among *Paenibacillus* strains isolated in this study.

Fig. S14 Biological assay for the isolated Paenibacillus strains.

Fig. S15 The *in vitro* assay to evaluate (a)(b) nitrogen (N_2) fixation and (c)(d) nitrous oxide (N_2O) generation from from the soil.

Fig. S16 Degree of detection in 2-aminoadipate and lysine leaves and roots and their ratio in root per leaf.

Fig. S17 Photos of cucumber roots in a field where knot-damages to plant parasitic nematode has been observed

Table contents

Table S1 Statistical values of the structural equation model candidates for the metabolite attributes and antioxidant activity of the leaf and root.

Table S2 Statistical values of the structural equation model candidates for the characteristic metabolites of the leaf and root.

Table S3 Statistical values of the structural equation model candidates for the metabolites and bacteria of the soil.

Table S4 A list of models targeted by causal mediation analysis for Fig. 5a and their statistical values.

Table S5 A list of models targeted by causal mediation analysis for Fig. 5b and their statistical values.

Table S6 A list of models targeted by causal mediation analysis for Fig. 5c and their statistical values.

Table S7 Nif-related genes identified based on the genomic data of *Paenibacillus macerans* HMSSN-036.

Table S8 Nif-related genes identified based on the genomic data of *Paenibacillus* sp. HMSSN-139.

Table S9 Other functional genes identified based on genomic data of *Paenibacillus macerans* HMSSN-036.

Table S10 Other functional genes identified based on the genomic data of *Paenibacillus* sp. HMSSN-139.

Table S11 Physicochemical indicators in the soil after cultivation of carrots.

Table S12 Statistical values of the structural equation model candidates for amino acids of the leaf and root.

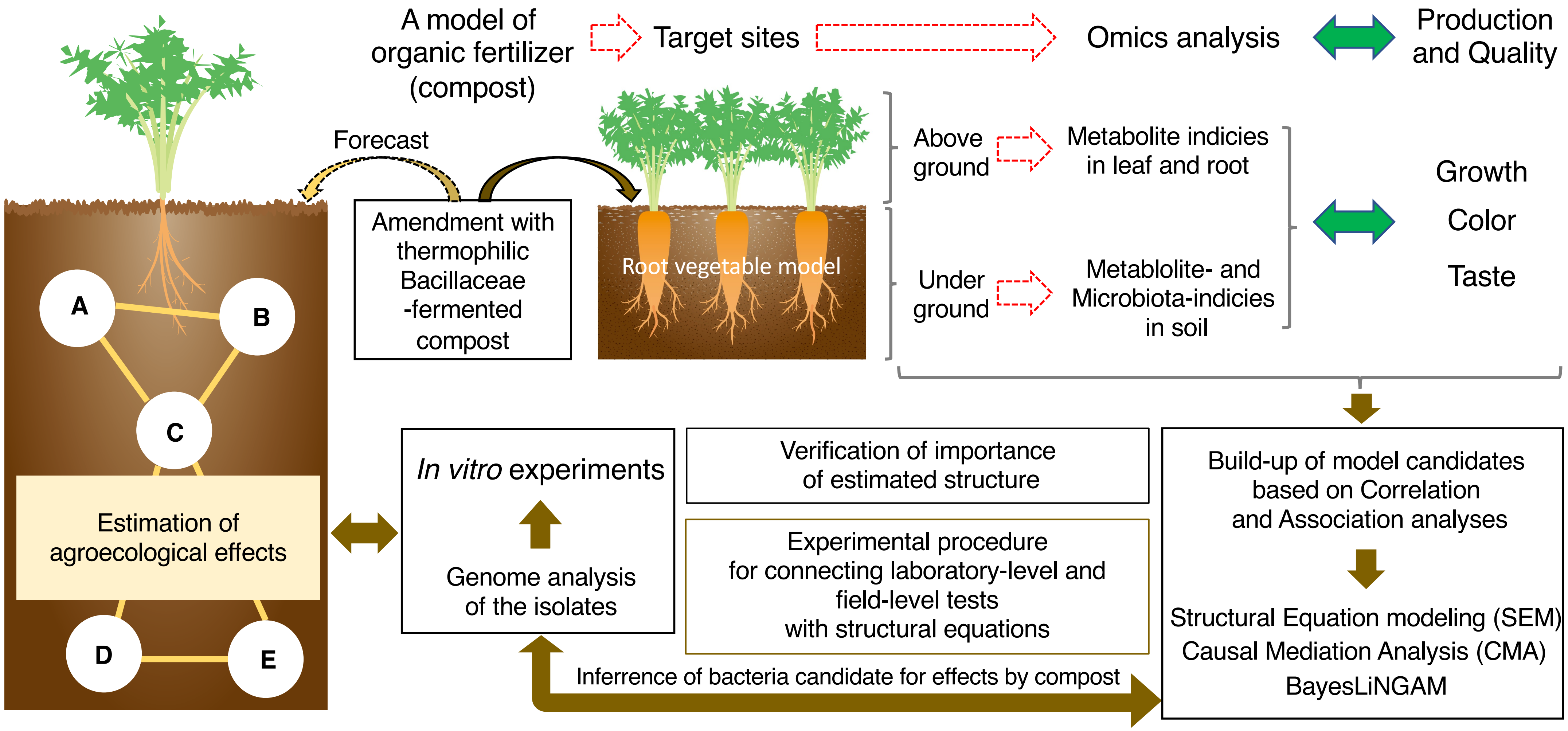

Fig. S1

Experimental scheme in this study. As a model of organic fertilizer, thermophile fermented compost was used, and as a crop model, carrots, which are easy to analyze both above and below the ground, were targeted. The leaves and roots, as well as soil metabolites, were analyzed, and the soil flora was analyzed to evaluate its relationships with growth, color, and taste as agricultural quality indices. These relationships were evaluated by correlation and association analysis, hypotheses were made, and a structural equation modeling (SEM) was constructed. In this study, since one of the bacterial candidates for nitrogen cycle was selected from the structural equation for the soil, the candidate bacteria were isolated, and their genomes were analyzed. Nitrogen fixation and suppression of nitrous oxide (N_2O) generation was also analyzed at the laboratory level. An analysis of the suppression of N_2O generation, which is difficult to perform in the field, was conducted in the laboratory. This study was performed using a procedure that connects laboratory-level and field-level tests with SEM to support experiments that cannot be conducted. In addition, causal mediation analysis (CMA) and BayesLiNGAM verified the importance as a group of optimal model candidates estimated by SEM. Based on these observations, a model for the agroecological effects of compost was estimated.

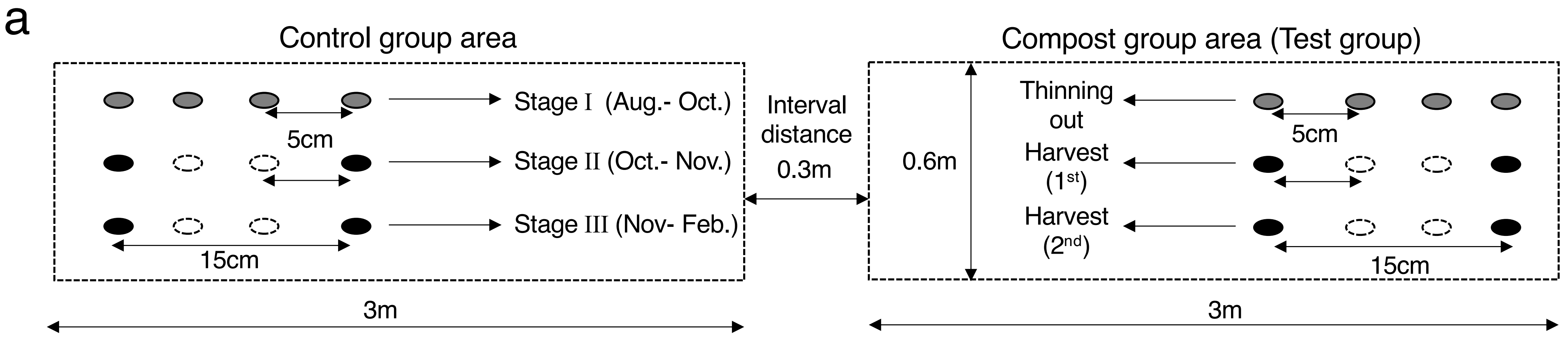

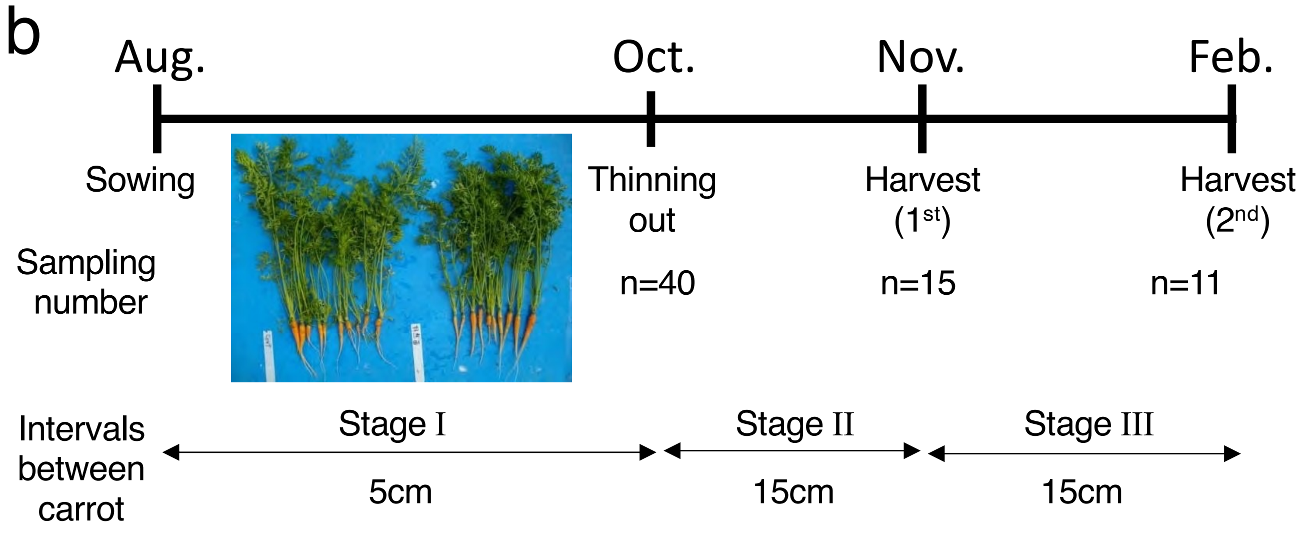

c

n=40	Height (cm)	Whole weight (g)	Root weight (g)	Stem & leaf weight (g)
Control	33.2 ± 5.6	9.8 ± 6.2	3.3 ± 2.4	6.4 ± 3.9
Compost	30.1 ± 4.2**	7.4 ± 3.9*	2.2 ± 1.6*	5.2 ± 2.5#

Fig. S2

Conditions for setting test conditions. (a) The relationship between the area and the distance of the field trial. Two growing areas of 0.6 m ridge width and 3 m ridge length were prepared and well agitated by a skilled person to ensure that no differences occurred. Next, a 0.3 m trench was set between the two groups and adjusted to avoid water exchange. Fertilizer was applied 8 days prior to planting. In the compost group area, compost powder was mixed. The seeds were then sown in three rows with a spacing of 5 cm between plants. In Stage I, planting was done at 5 cm spacing, and the plants were planted in August 2016. Subsequently, thinning was conducted in October 2016, and the plant spacing was adjusted to 15 cm (Stage II). The first harvest was conducted in November 2016. As Stage III, cultivation was continued and the second harvest was conducted in February 2017. Procedures within the dotted square are performed simultaneously by all rows in the group. (b) A schedule is illustrated to show the number of samplings and plant spacing. The photo shows the thinned carrots. (c) Data showing growth surveys conducted in October on thinned carrots are shown. Significant values were shown as follows: **, $p < 0.01$; *, $p < 0.05$; #, $p < 0.1$.

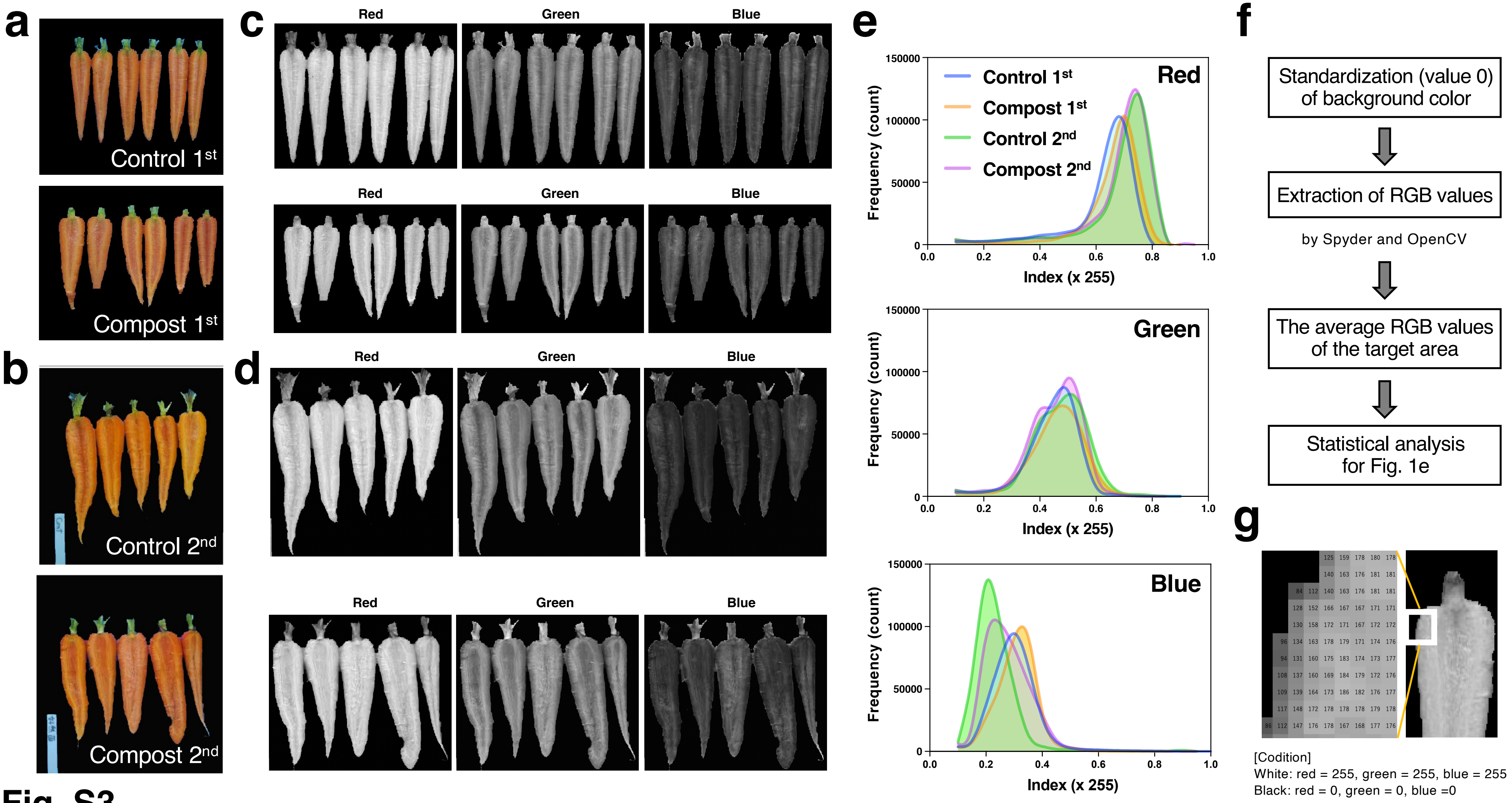

Fig. S3

Pre-processing for image analysis. In brief, the background of the photos was converted to black. The following figure shows the converted backgrounds of photos of (a) carrots harvested in November and (b) carrots harvested in February. The photo of (a) and (b) were transformed to grayscale photo (c) and (d) with RGB color indices. (e) The count profiles for RGB colour calculated by the R software library 'imager' was visualized by Prism software. Next, Fig. 1e was analyzed according to Fig. 1d and (f) the procedure described in the Supplementary Method. (g) RGB values were calculated for arbitrarily divided pixels, as indicated by the white squares. The average of these numbers per individual carrot was calculated for the range shown in Fig. 1d, and statistical analysis was performed utilizing these averaged numbers. The abbreviations in the photo (a) and (b) indicate as follows: Control 1st, and Compost 1st, carrots of the control and compost group harvested in November 2016, respectively; Control 2nd, and Compost 2nd, carrots of those groups harvested in Febrary 2017, respectively.

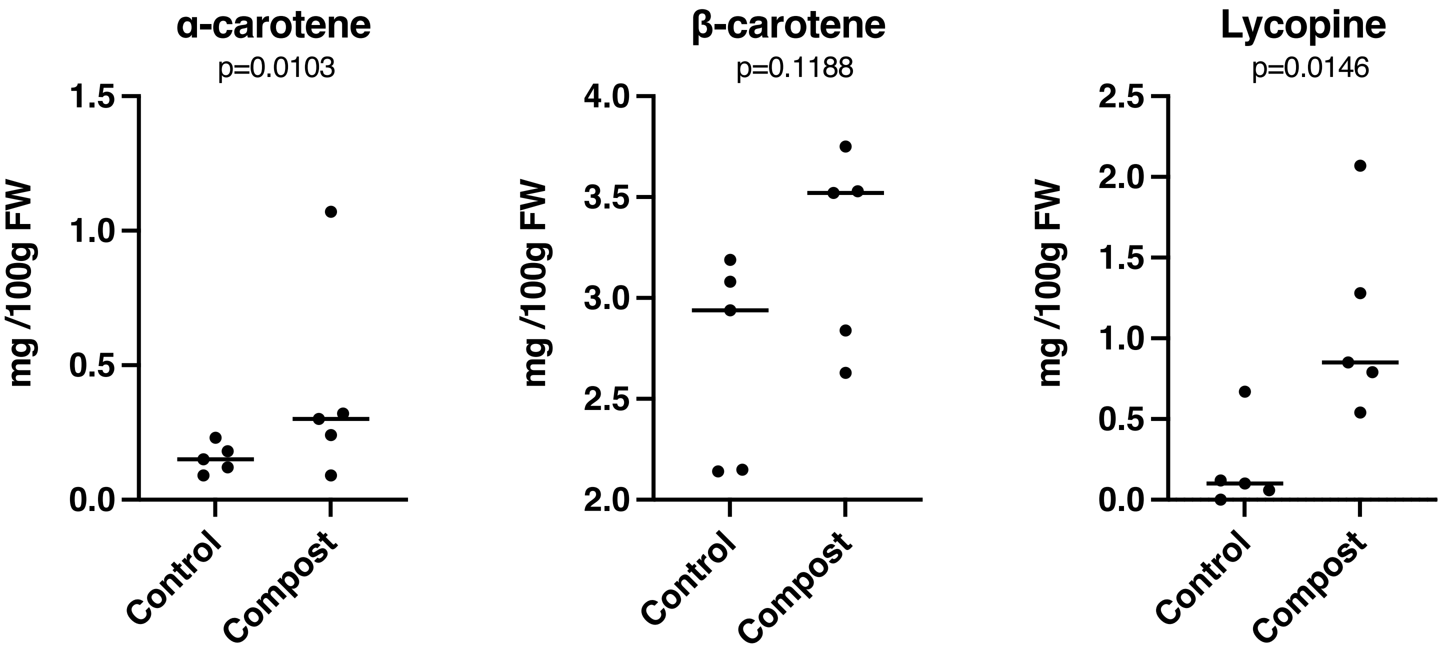

Fig. S4

The contents of primary carotenoids on the roots of carrots harvested in Febrary 2017. The content of α-carotene, β-carotene, and lycopene (n=5) was shown. The "Control" and "Compost" show the data under normal conditions (control group) and compost-amended conditions (compost group), respectively.

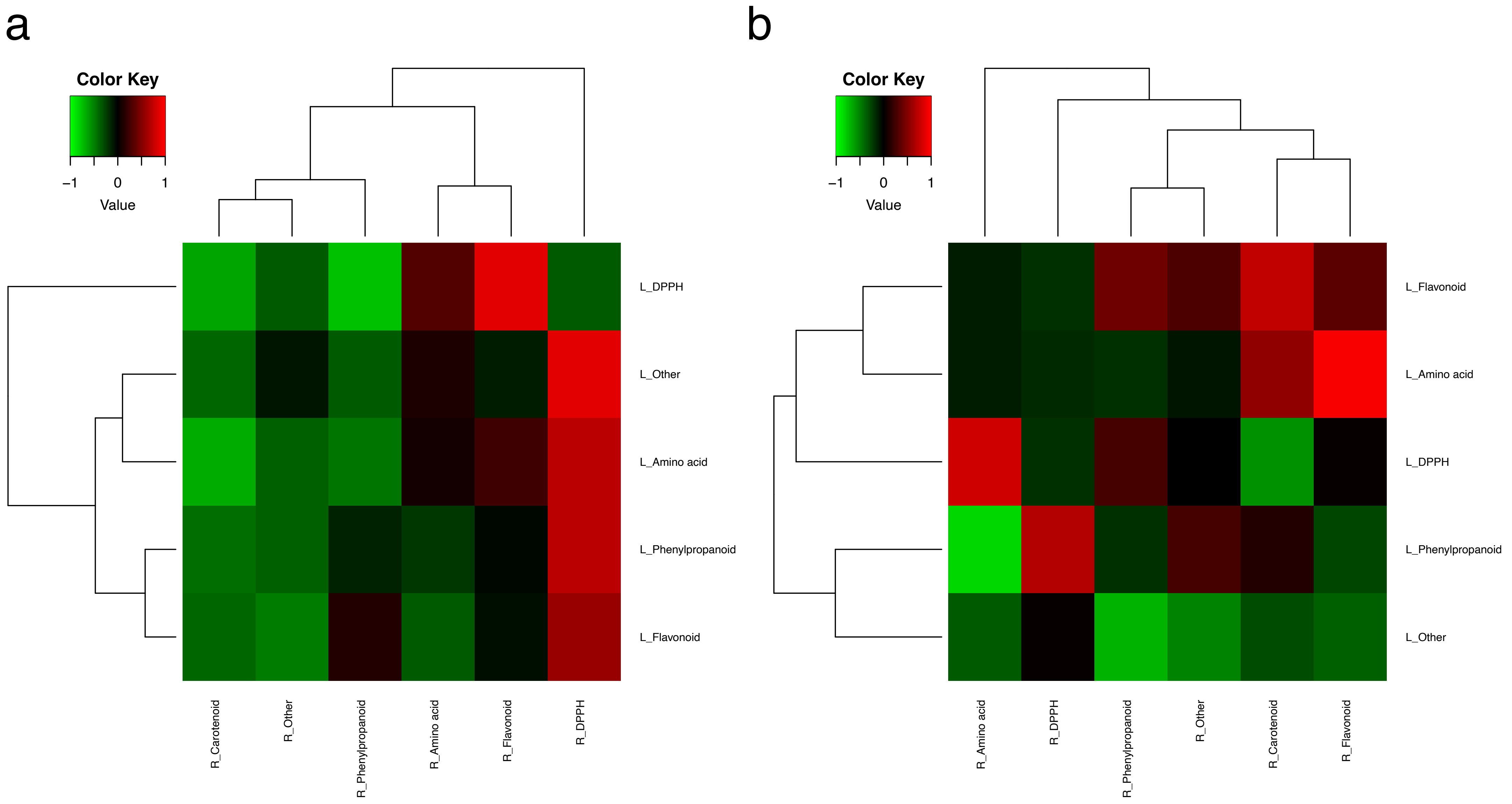

Fig. S5

Heatmaps of the correlations between the amino acid, carotenoid, flavonoid, and DPPH activity in the leaves and roots. The heatmaps based on data in (a) the control group and (b) the compost (Test) group were shown.

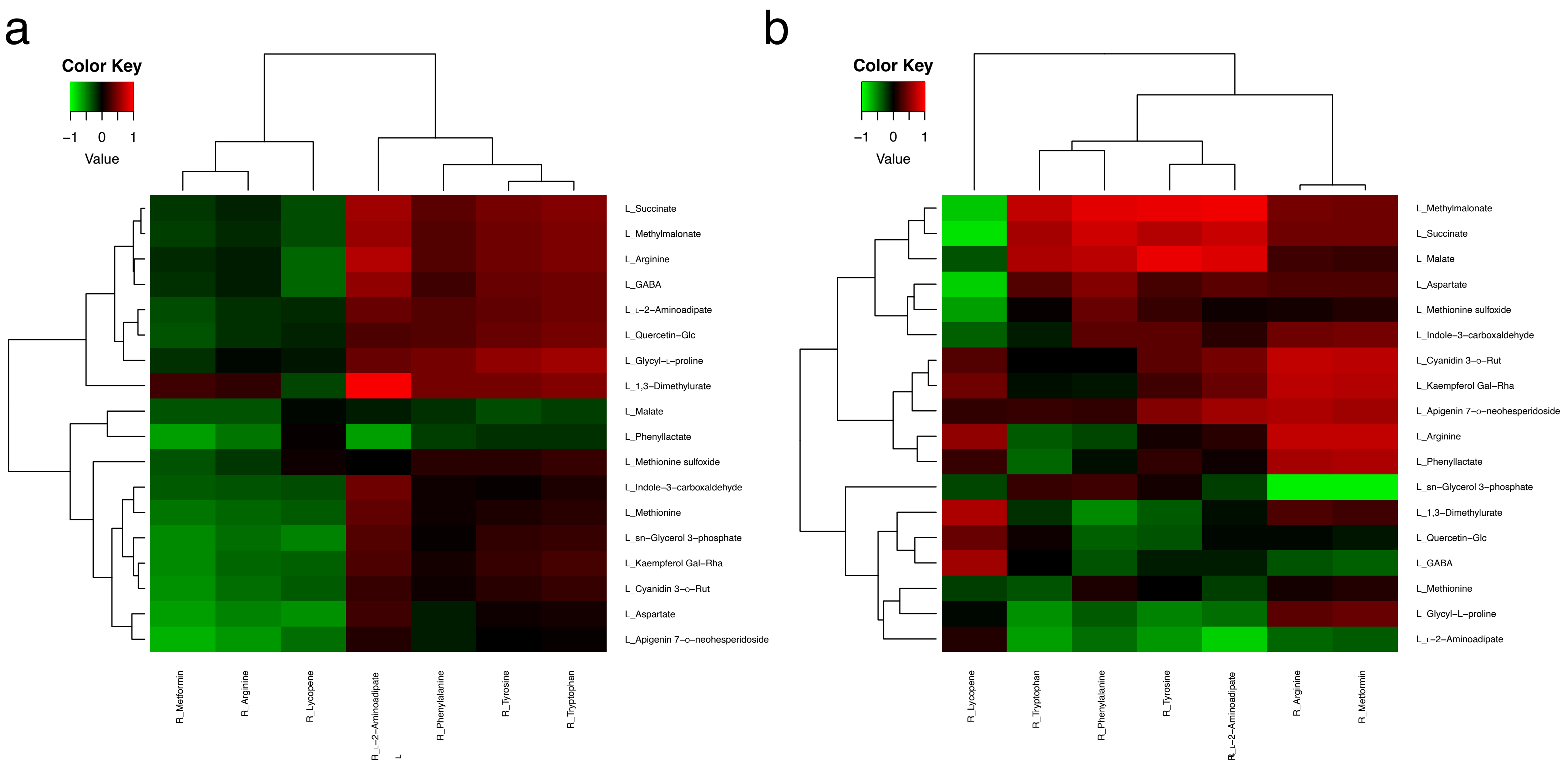

Fig. S6

Heatmaps of the correlations between metabolite candidates in the leaves and roots shown in Fig. 3. The heatmaps based on the data in (a) the control group and (b) the compost (Test) group were shown.

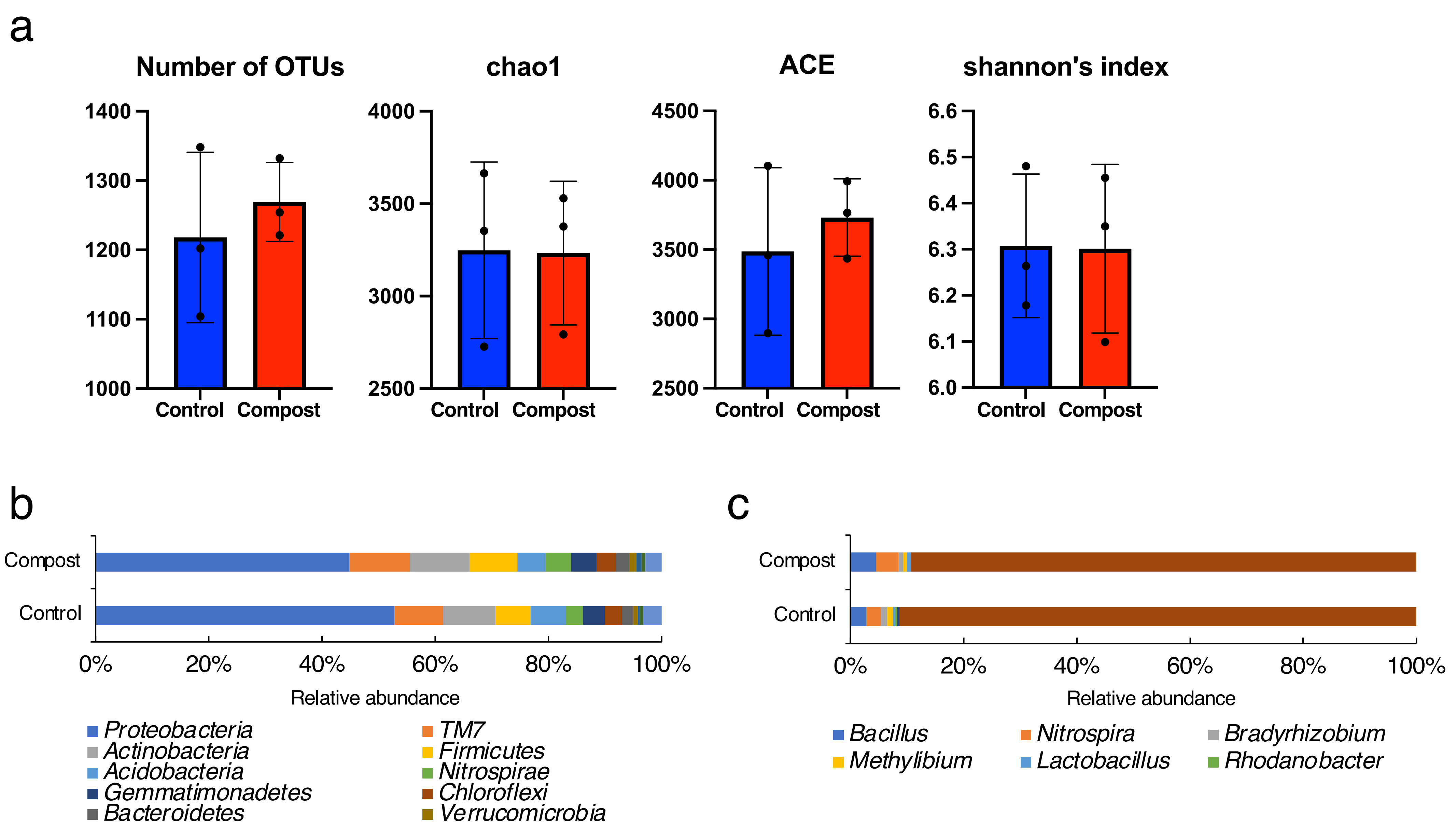

Fig. S7

Bacterial diversity in the soil after cultivation of carrot. (a) OTU numbers and Chao1, ACE, and Shannon indices representing α-diversity under normal conditions (Control) and compost-amended conditions (Compost). The bacterial population in the soil of the control and test groups showing as relative abundances of the (b) phyla and (c) genera (>1% as maximum of the bactereiral population).

a

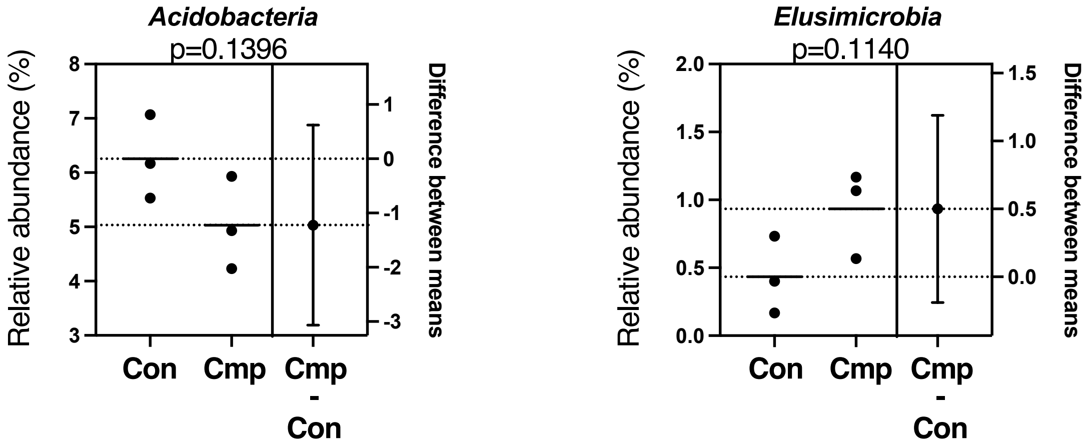

Fig. S8

The estimation plot of the bacterial population in the soil of the control and test groups. Relative abundances of the (a) phyla and (b) genera (0.1 < p <0.2; >0.1% as maximum of bacterial population). The abbreviations were shown as follows: Con, the control group; Cmp, the compost (test) group. Cmp-Con, the ratio of the compost group data for the control group data.

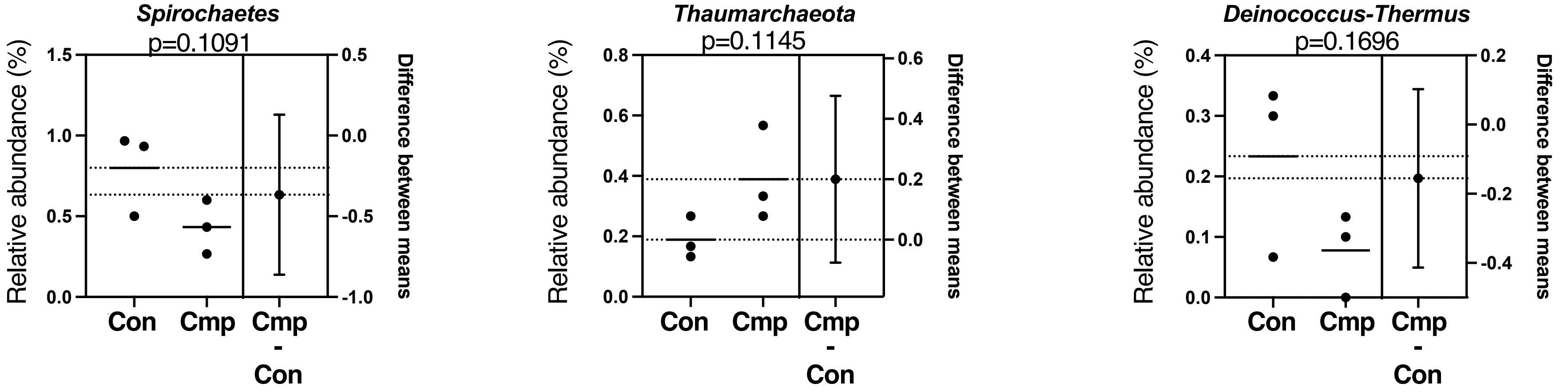

b

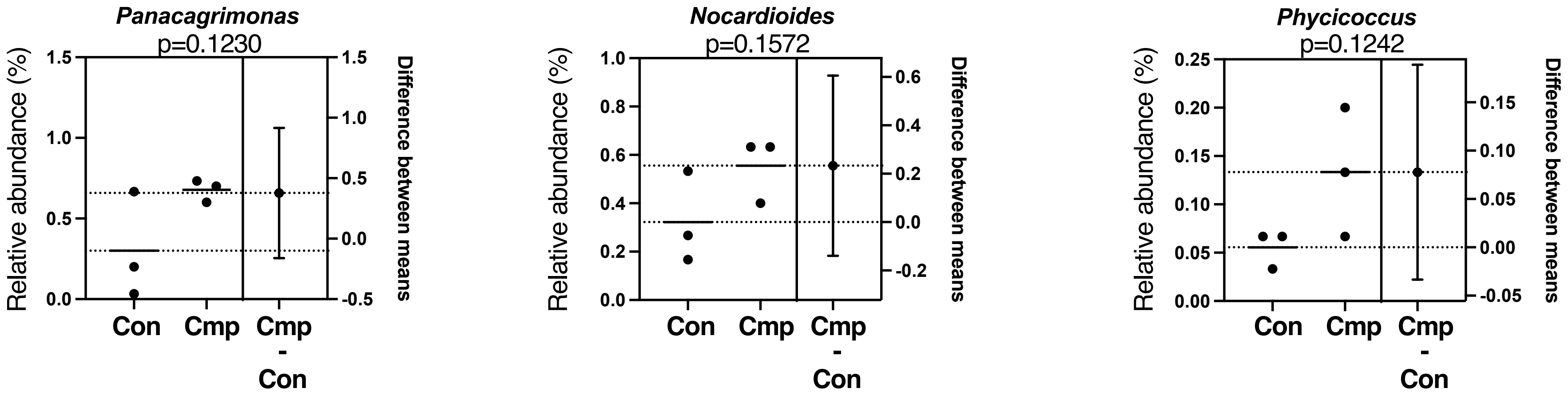

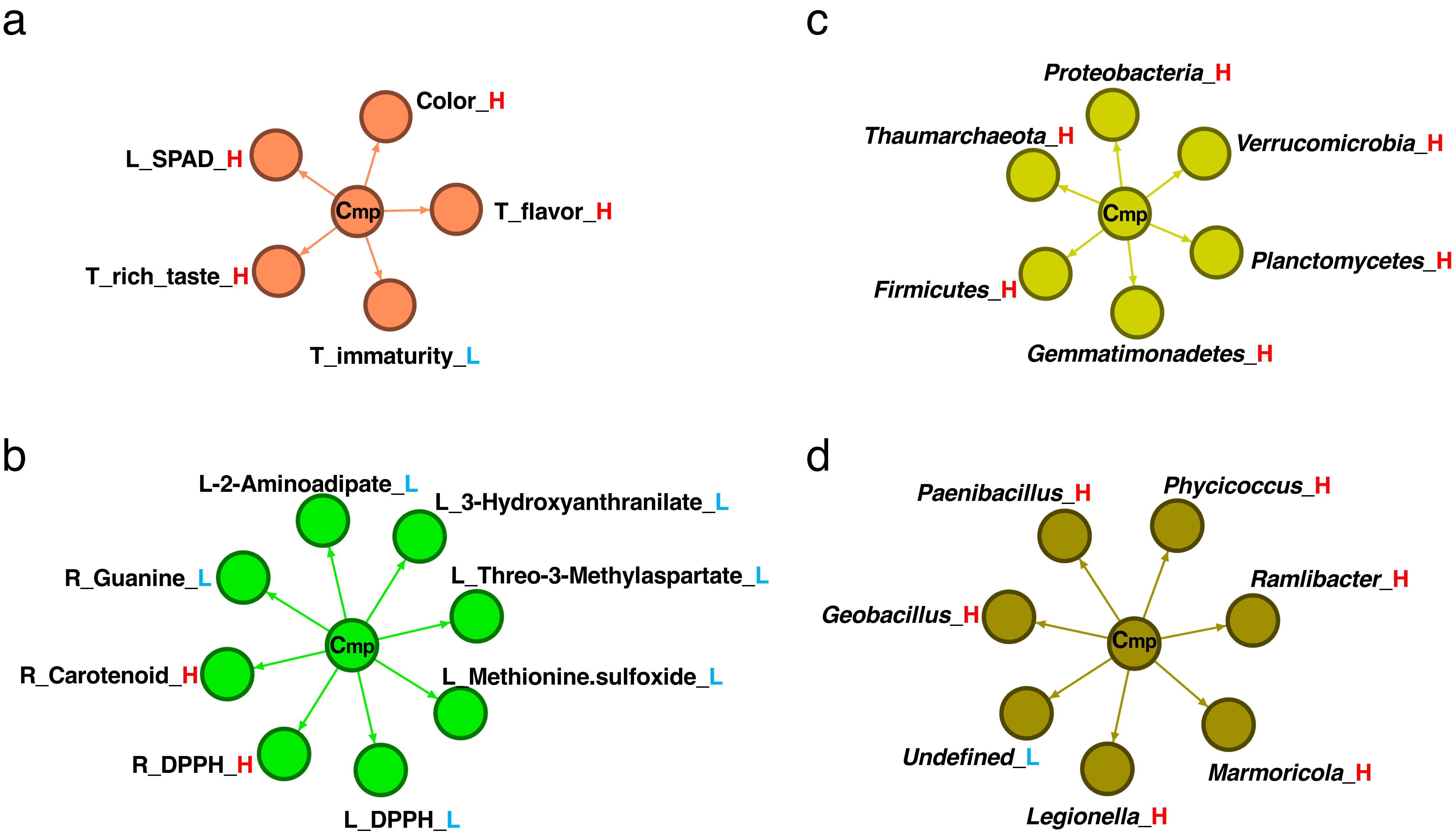

Fig. S9

Association network in (a) appearance and taste, (b) leaves and root metabolites, (c) soil bacterial phyla and (d) genera (lift values > 2.0). The abbreviations were shown as follows: Cmp, the compost (test) group. H (red color), High; L (blue color), Low; L (black color), leaf; R, root; T, Taste.

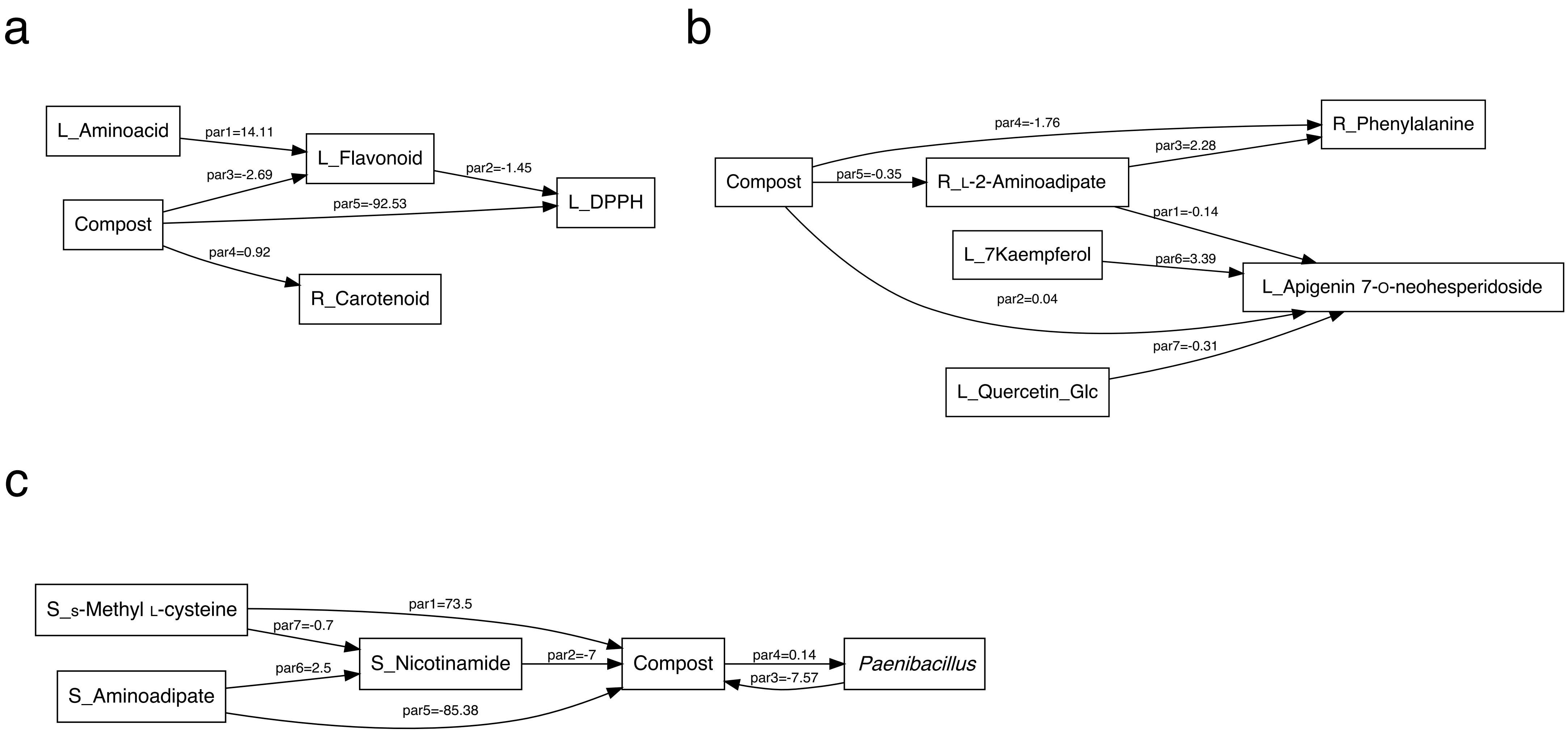

Fig. S10

The path of Fig. 5 calculated by the function “sem”. (a), (b), and (c) shows the path in Figs. 5a, 5b, and 5c, respectively. The value in the back of each “par=” shows the “startvalue” as the parameter (par) of the indicated each path. The number in back of each “par=” shows just a simple order of description.

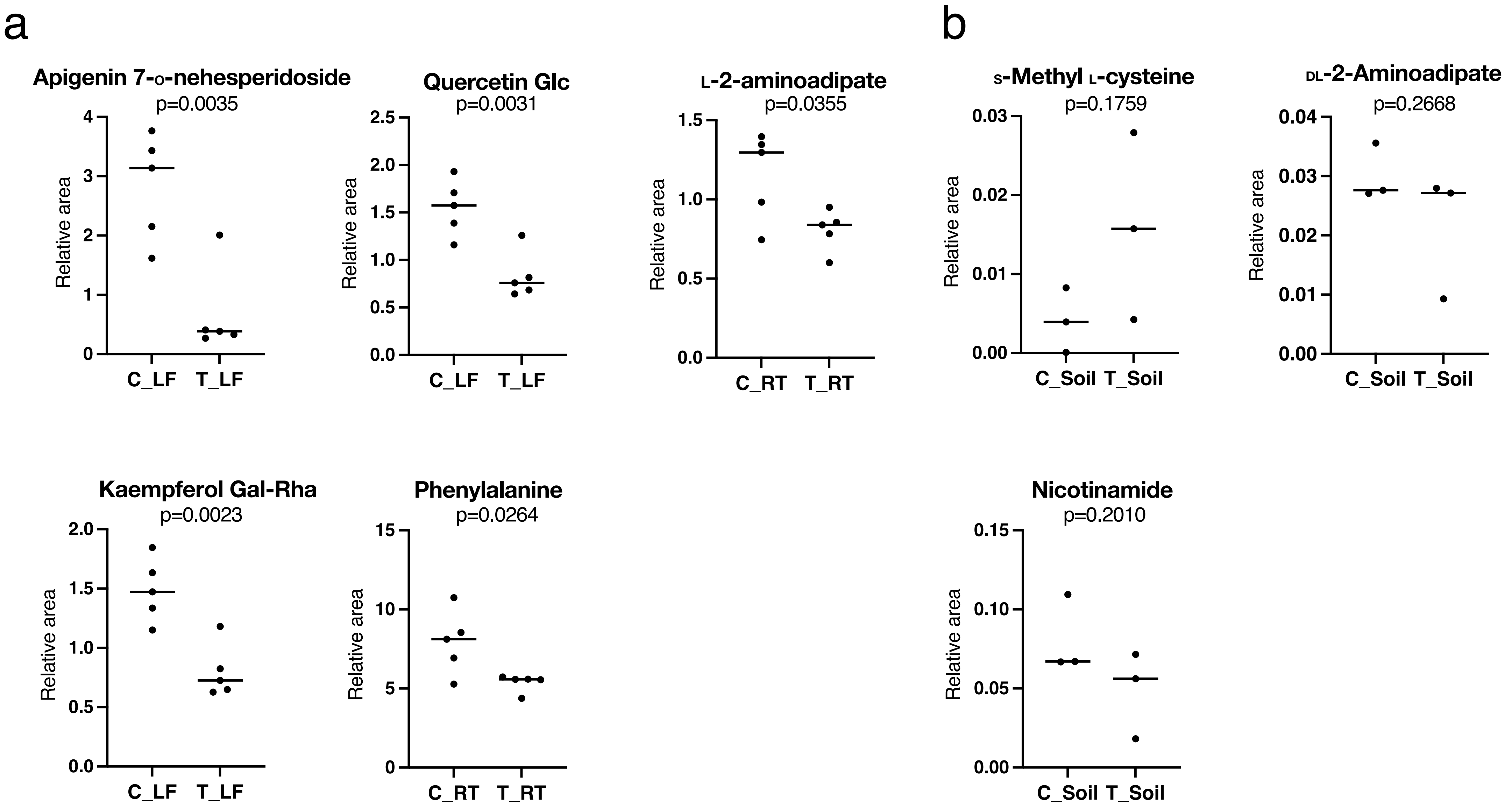

Fig. S11

Degree of detection in metabolite candidates used for optimal structural equations. (a) The relative area of metabolite candidates in Fig. 5b. (b) The relative area of metabolite candidates in Fig. 5c. The abbreviations were shown as follows: C_LF, leaf in control group; T_LF, leaf in compost group (Test group); C_RT, root in the control group; T_RT, root in the compost (Test) group; C_Soil, soil in the control group; T_Soil, soil in the compost (Test) group.

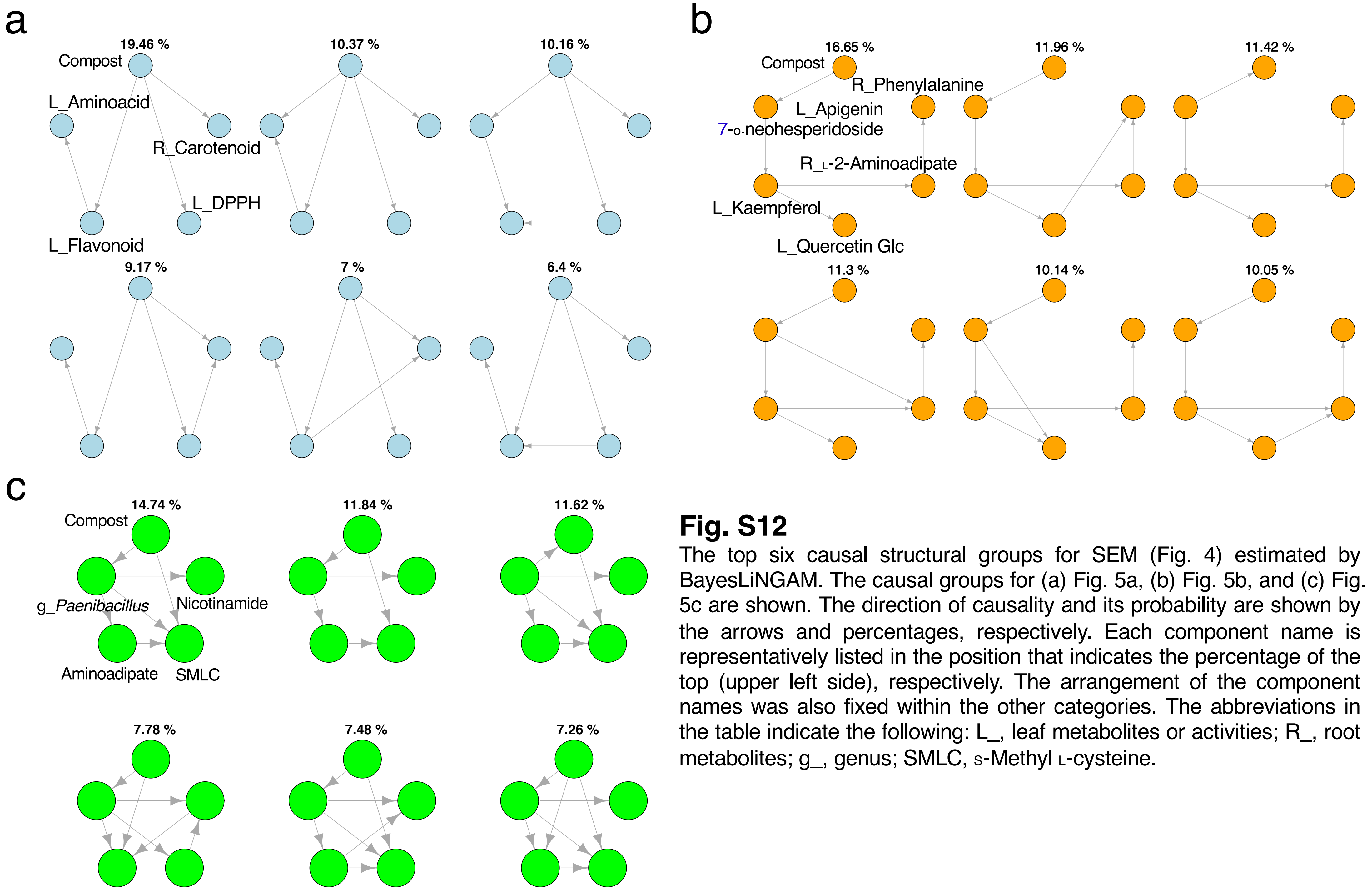

Fig. S12

The top six causal structural groups for SEM (Fig. 4) estimated by BayesLiNGAM. The causal groups for (a) Fig. 5a, (b) Fig. 5b, and (c) Fig. 5c are shown. The direction of causality and its probability are shown by the arrows and percentages, respectively. Each component name is representatively listed in the position that indicates the percentage of the top (upper left side), respectively. The arrangement of the component names was also fixed within the other categories. The abbreviations in the table indicate the following: L_, leaf metabolites or activities; R_, root metabolites; g_, genus; SMLC, S-Methyl L-cysteine.

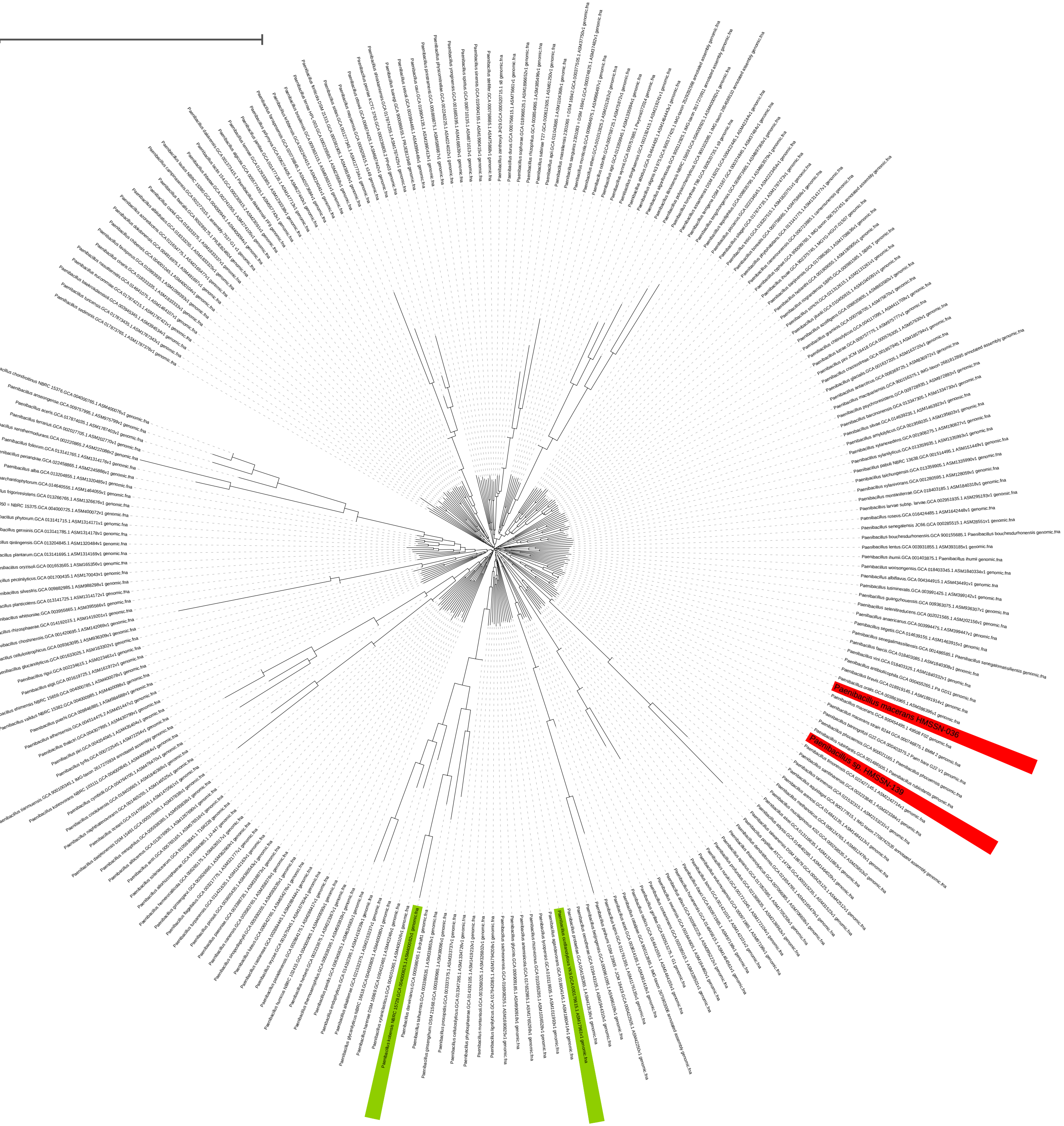

Fig. S13

Phylogenetic relationship among *Paenibacillus* strains isolated in this study. The whole genome phylogenetic analysis of the isolated strains was performed based on the mash distance. The phylogenetic tree was constructed using the neighbor-joining method. The isolated strains are marked in red color. Species detected by 16SrRNA analysis of soil are marked in green.

a

+: positive reaction

strain	IAA productivity	phosphate solubilization	siderophore reaction
#36	+	+	+
#139	+	+	+

b

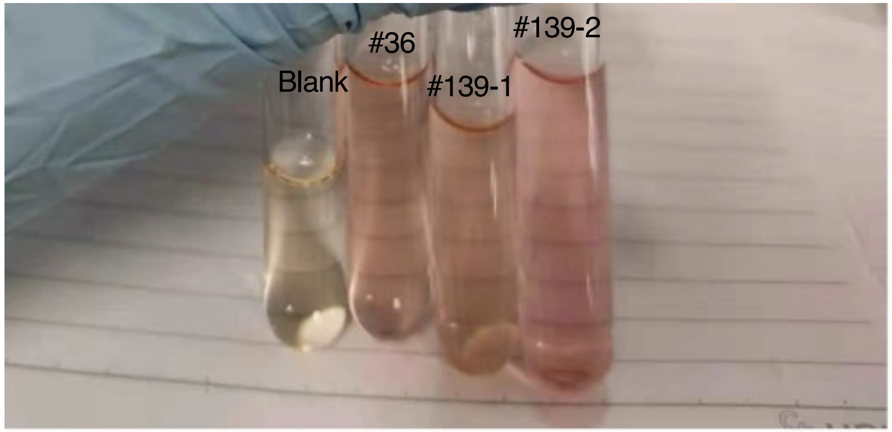

c

Strain	Before King B cultivation	48hr after King B cultivation (Before measurement)		IAA conc. (μg/ml)
	Initial conc. (CFU / ml) in King B broth	Dilution	OD600	
#36	4.80E+04	1	0.423 ± 0.024 (n=3)	47.42 ± 2.73 (n=3)
#139	2.96E+02	5	0.403 ± 0.011 (n=3)	54.75 ± 4.89 (n=3)

d

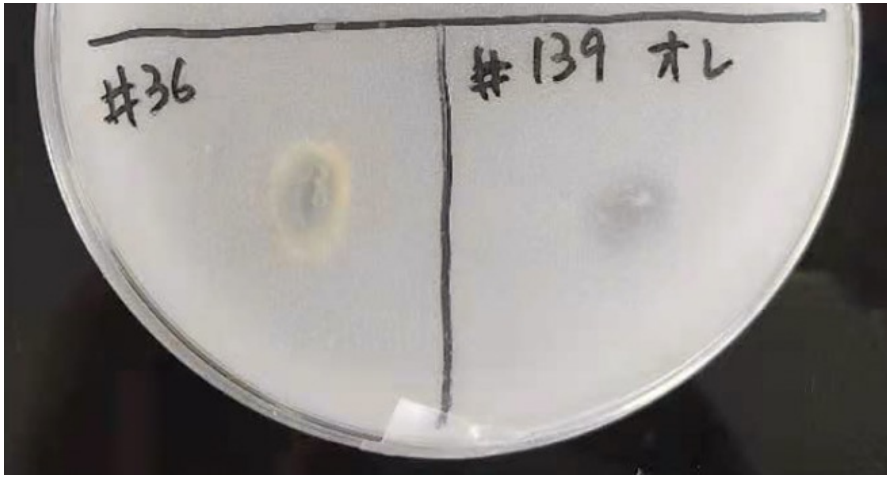

e

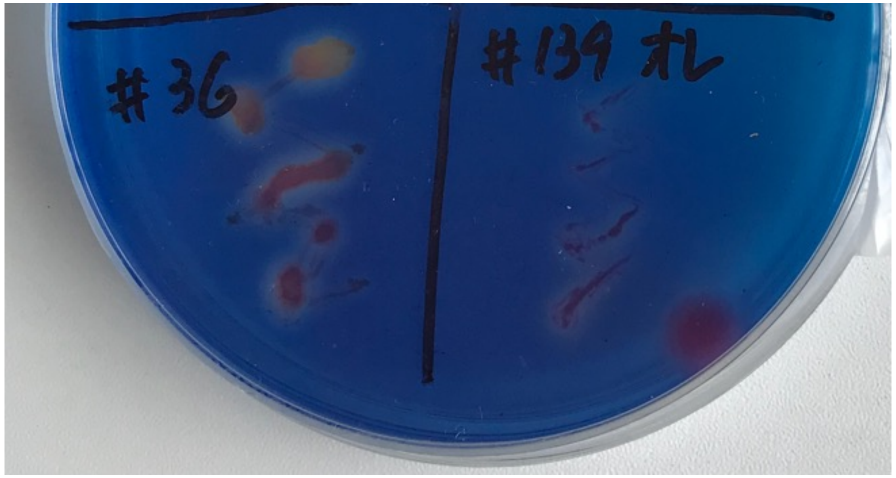

Fig. S14

Biological assay for isolated *Paenibacillus* strains. (a) Summarized data from the biological assay. (b)-(d) shows the photo of the assay. The names in the photo show blank (a sample without the bacteria), the strain number of different cultivating samples, respectivley. (b) The photos were visualized the potential ability of the IAA productivity based on the alteration of liquid color. (c) The contents of these samples were measured based on the calibration curves ($y = 115.94x$, R^2=0.9965 for #36; $y = 122.13x$, R^2=0.9711 for #139). (d) phosphate solubilization, and (e) siderophore reaction tests of selected strains are shown.

a

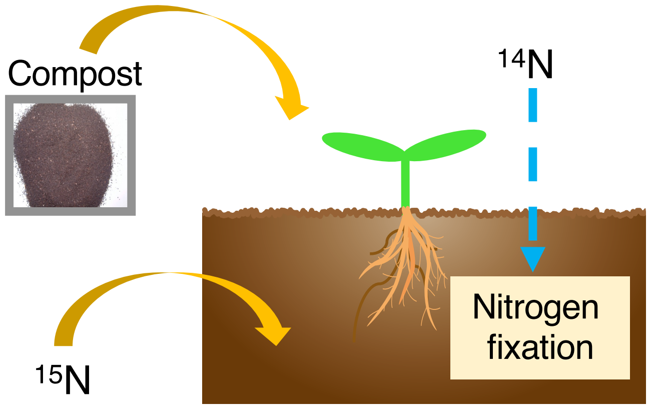

Fig. S15

In vitro assay to evaluate (a)(b) nitrogen (N_2) fixation and (c)(d) nitrous oxide (N_2O) generation from from the soil. (a) Conceptual diagram of the test model with the stable isotope ^{14}N is shown. The experiments to evaluate nitrogen fixation were carried out in the test model. (b) Biological weight of plants in the stable isotope test. The ratios of the ^{14}N and the stable isotopes ^{15}N contained in freeze-dried crop and soil samples are shown. S-Compost, the group to which sterile compost was added; Compost, the group to which compost was added; and Compost (60 °C), the group to which high temperature treated compost was added immediately before the experiment. (c) The conceptual diagram of the test model is shown. After a container containing soil was inserted into Gas package I, it was joined to Gas package II with a cock. These sets were prepared with different soil conditions. The composition of fungi in the prepared preexperimental soil was also investigated. (d) shows the N_2O concentration per 2 hours generated from the soil. The "Soil only" indicates the soil only group. PD indicates potato dextrose. The contents of N_2O from "Soil" and "Soil + Compost" groups were markedly different ($p < 0.1$).

b

Category	Plant				Soil	
	FW	n	$\delta^{15}N$ (‰)	^{15}N / ^{14}N	$\delta^{15}N$ (‰)	^{15}N / ^{14}N
Soil only	0.832 ± 0.134	3	4.9	0.051	4.16	0.043
Soil + S-Compost	1.419 ± 0.348	3	4.68	0.049	3.18	0.033
Soil + Compost	1.608 ± 0.407	3	3.92	0.041	2.74	0.028
Soil + Compst (60°C)	1.252 ± 0.161	3	3.95	0.041	3.09	0.032

c

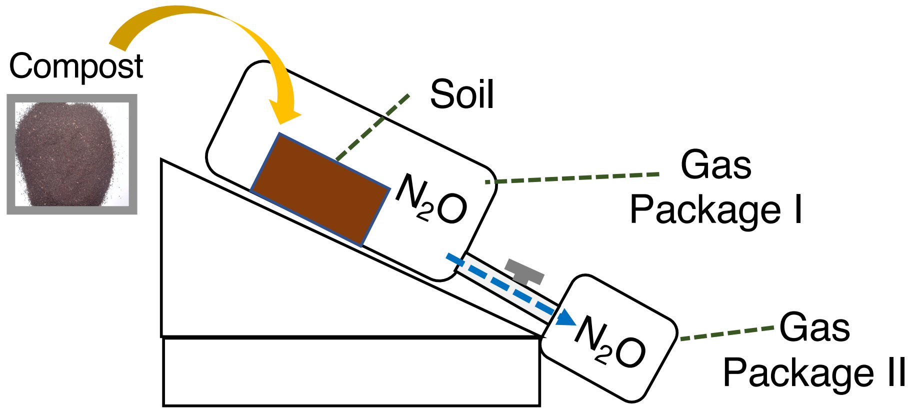

d

Category	PDA	n	N_2O (ppb)	p value
Soil (PDA-)	-	1	120.31	-
Soil only	+	3	145.20 ± 10.37	-
Soil + Compost	+	3	119.59 ± 5.07	0.0684

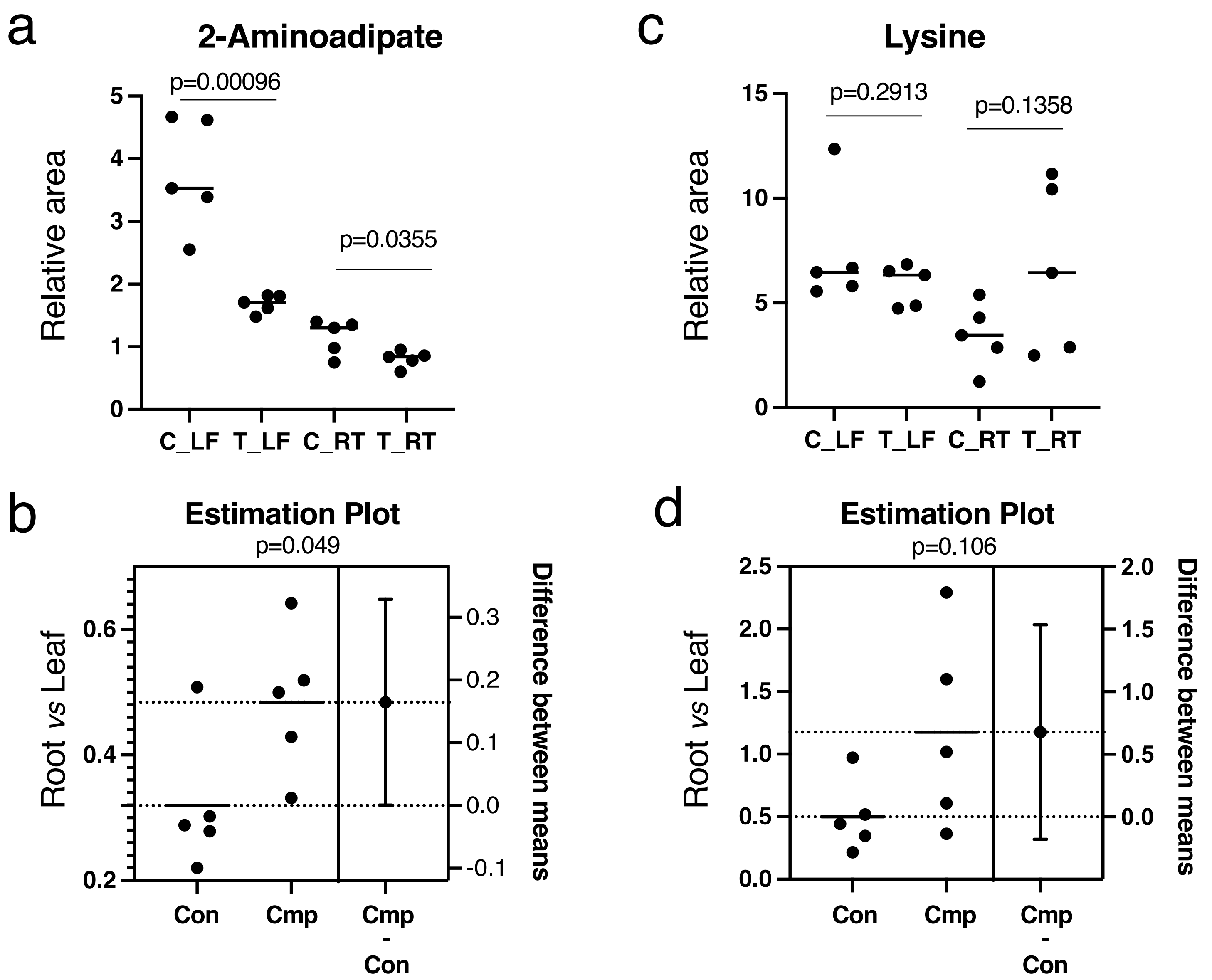

Fig. S16

Degree of detection in leaves and roots of 2-aminoadipate and lysine and their ratio in root per leaf. (a) shows the relative area of 2-aminoadipate standardized by the internal standard. (b) shows the ratio of 2-aminoadipate in the root per leaf. (c) shows the relative area of lysine standardized by the internal standard. (d) shows the ratio of lysine in root per leaf. The abbreviations were shown as follows: C_LF, leaf in control group; T_LF, leaf in compost group (Test group); C_RT, root in the control group; T_RT, root in the compost group (Test group); Con, the control group; Cmp, the compost group.

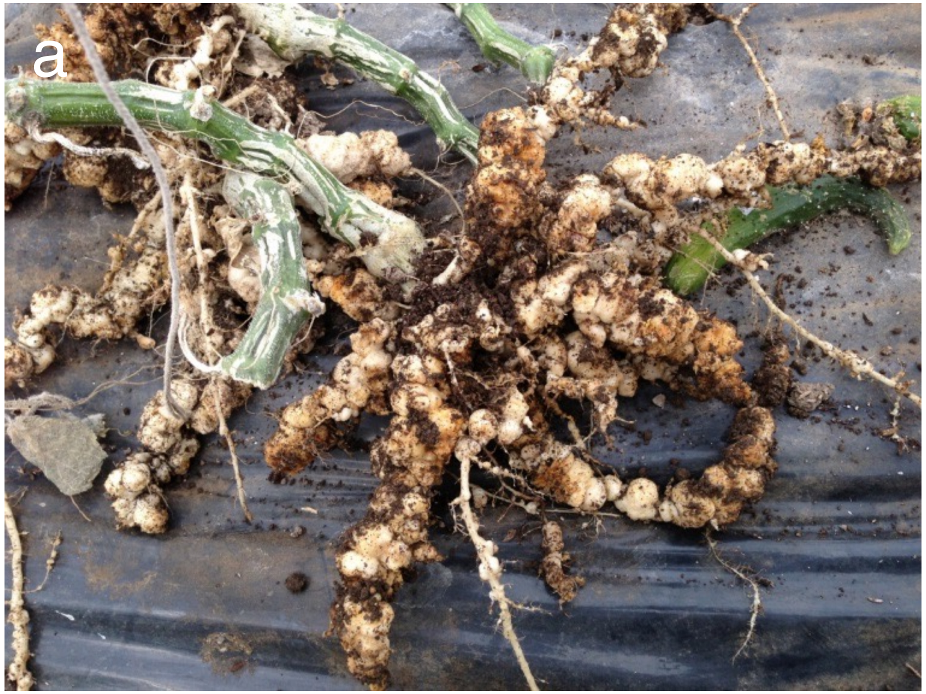

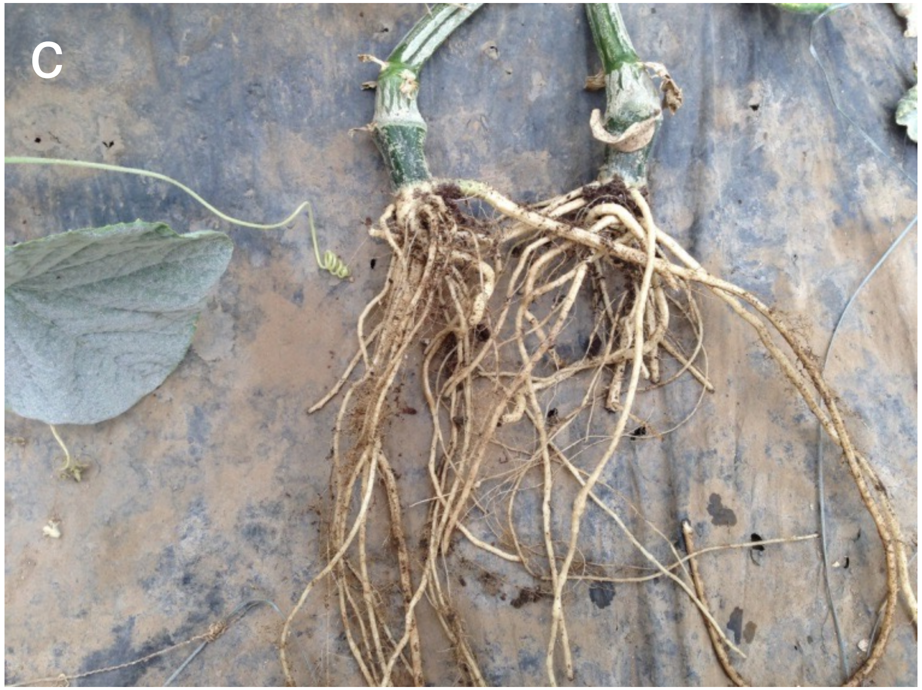

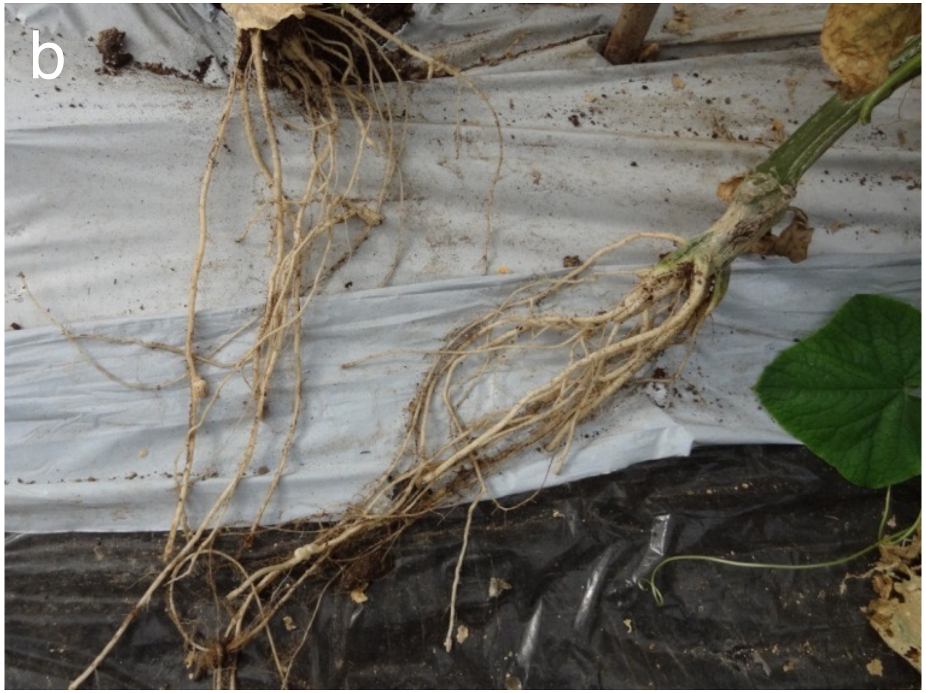

Fig. S17

Photos of cucumber roots in a field where damage to the plant parasitic nematode has been observed: (a) roots with several humps infected with the nematode in 2010 (b) roots infected with the nematode but with a few humps in 2011 (c) roots with fewer humps even though the nematodes were present in the soil in 2014. In the field, a 100-fold diluent of thermophile-fermented compost was added to the soil after 2010. The diluent was irrigated in the soil at least once every two weeks during the cultivation period.

Table S1

Statistical values of the final optimal structural equation models for the metabolite attributes and antioxidant activity of the leaf and root.

Category	Model	Fit indices		
	L_Flavonoid ~ L_Aminoacid	chisq 2.061	df 4.000	pvalue 0.725
	L_DPPH ~ L_Flavonoid	cfi 1.000	tli 1.128	rfi 0.893
No.1	L_Flavonoid + R_Carotenoid + L_DPPH ~ Compost	nfi 0.952	srmr 0.033	AIC 145.889
	lavaan 0.6-11 ended normally after 2 iterations	rmsea 0.000	gfi 0.997	agfi 0.988
	Number of successful bootstrap draws 909			
	L_Flavonoid ~ L_Aminoacid	chisq 2.115	df 5.000	pvalue 0.833
	L_Flavonoid + R_Carotenoid + L_DPPH ~ Compost	cfi 1.000	tli 1.152	rfi 0.912
No.2		nfi 0.951	srmr 0.038	AIC 143.943
	lavaan 0.6-11 ended normally after 1 iterations	rmsea 0.000	gfi 0.996	agfi 0.989
	Number of successful bootstrap draws 939			
	Compost + L_Flavonoid ~ L_Aminoacid	chisq 11.835	df 8.000	pvalue 0.159
	L_Other ~ L_Flavonoid	cfi 0.936	tli 0.879	rfi 0.702
No. 3	L_Flavonoid + R_Carotenoid + L_Other + L_DPPH ~ Compost	nfi 0.841	srmr 0.049	AIC 196.609
	lavaan 0.6-11 ended normally after 2 iterations	rmsea 0.219	gfi 0.853	agfi 0.613
	Number of successful bootstrap draws 719			

The abbreviations in the table indicate the following: L_, leaf; R_, root; chisq, Chi-square: χ^2; df, degrees of freedom (DF); p-value, p values (Chi-square); cfi, comparative fix index (CFI); tli Tucker–Lewis index (TLI); nfi, (non) normed fit index; rfi, relative fit index (RFI); srmr, standardized root mean residuals (SRMR); AIC, Akaike information criterion; rmsea, root mean square error of approximation (RMSEA); gfi, goodness-of-fit index (GFI); and agfi, adjusted goodness-of-fit index (AGFI). Column No. 1 shows the best numerical structural equation model (Fig. 5a). Column No. 2 shows the inferior numerical structural equation model. Column No. 3 shows another inferior numerical structural equation model.

Table S2

Statistical values of the optimal structural equation model candidates for the characteristic metabolites of the leaf and root.

Category	Model	Fit indices		
	L_Apigenin_7_O_neohesperidoside + R_Phenylalanine ~ R_2_Aminoadipate + Compost	chisq 3.048	df 5.000	pvalue 0.693
	R_2_Aminoadipate ~ Compost	cfi 1.000	tli 1.112	rfi 0.864
	L_Apigenin_7_O_neohesperidoside ~ L_Kaempferol + L_Quercetin_Glc	nfi 0.943	srmr 0.054	AIC 45.064
No1		rmsea 0.000	gfi 1.000	agfi 0.999
	lavaan 0.6-11 ended normally after 1 iterations			
	Number of successful bootstrap draws 640			
	L_Apigenin_7_O_neohesperidoside + R_Phenylalanine ~ R_2_Aminoadipate + Compost	chisq 2.857	df 6.000	pvalue 0.827
	R_2_Aminoadipate ~ Compost	cfi 1.000	tli 1.141	rfi 0.899
	L_Apigenin_7_O_neohesperidoside ~ L_Kaempferol + L_Quercetin_Glc	nfi 0.957	srmr 0.046	AIC 51.102
No2	Compost ~ L_Apigenin_7_O_neohesperidoside	rmsea 0.000	gfi 1.000	agfi 0.999
	lavaan 0.6-11 ended normally after 33 iterations			
	Number of successful bootstrap draws 637			
	L_Apigenin_7_O_neohesperidoside + R_Phenylalanine ~ R_2_Aminoadipate + Compost	chisq 2.085	df 3.000	pvalue 0.555
	R_2_Aminoadipate ~ Compost	cfi 1.000	tli 1.055	rfi 0.893
	L_Apigenin_7_O_neohesperidoside ~ L_Kaempferol	nfi 0.968	srmr 0.041	AIC 49.316
No3	Compost ~ L_Apigenin_7_O_neohesperidoside	rmsea 0.000	gfi 1.000	agfi 0.999
	lavaan 0.6-11 ended normally after 32 iterations			
	Number of successful bootstrap draws 916			
	L_Apigenin_7_O_neohesperidoside + R_Phenylalanine ~ R_2_Aminoadipate + Compost	chisq 0.241	df 1.000	pvalue 0.624
	R_2_Aminoadipate ~ Compost	cfi 1.000	tli 1.229	rfi 0.944
No4		nfi 0.991	srmr 0.018	AIC 66.167
	lavaan 0.6-11 ended normally after 1 iterations	rmsea 0.000	gfi 0.996	agfi 0.960
	Number of successful bootstrap draws 992			

The abbreviations in the table indicate the following: L_, leaf; R_, root; chisq, Chi-square: χ^2; df, degrees of freedom (DF); p-value, p values (Chi-square); cfi, comparative fix index (CFI); tli Tucker–Lewis index (TLI); nfi, (non) normed fit index; rfi, relative fit index (RFI); srmr, standardized root mean residuals (SRMR); AIC, Akaike information criterion; rmsea, root mean square error of approximation (RMSEA); gfi, goodness-of-fit index (GFI); and agfi, adjusted goodness-of-fit index (AGFI). Column No. 1 shows the best numerical structural equation model (Fig. 5b). Column No. 2 shows the inferior numerical structural equation model. Column No. 3 shows another inferior numerical structural equation model.

Table S3

Statistical values of the final optimal structural equation model candidates for the metabolites and bacteria of soil.

Category	Model	Fit indices		
No1	Compost ~ S_s_Methyl L_cysteine + S_Nicotinamide + *Paenibacillus*	chisq 0.989	df 2.000	pvalue 0.61
	Paenibacillus ~ Compost	cfi 1.000	tli 1.121	rfi 0.905
	Compost + S_Nicotinamide ~ S_Aminoadipate	nfi 0.979	srmr 0.005	AIC -58.057
	S_Nicotinamide ~ S_s_Methyl L_cysteine	rmsea 0.000	gfi 1.000	agfi 1.000
	lavaan 0.6-11 ended normally after 40 iterations			
	Number of successful bootstrap draws 24			
No2	Compost ~ S_s_Methyl L_cysteine + S_Nicotinamide + Paenibacillus	chisq 0.989	df 2.000	pvalue 0.61
	Paenibacillus ~ Compost	cfi 1.000	tli 1.101	rfi 0.918
	Compost ~ S_Aminoadipate	nfi 0.977	srmr 0.055	AIC -33.11
	lavaan 0.6-11 ended normally after 38 iterations	rmsea 0.000	gfi 1.000	agfi 1.000
	Number of successful bootstrap draws 42			
No3	Compost ~ S_s_Methyl L_cysteine + S_Nicotinamide + *Paenibacillus* + *Geobacillus*	chisq 1.128	df 2.000	pvalue 0.569
	Paenibacillus ~ Compost	cfi 1.000	tli 1.128	rfi 0.872
		nfi 0.963	srmr 0.049	AIC -21.697
	lavaan 0.6-11 ended normally after 21 iterations	rmsea 0	gfi 1.000	agfi 0.999
	Number of successful bootstrap draws 60			
No4	Compost ~ S_s_Methyl L_cysteine + S_Nicotinamide + *Paenibacillus*	chisq 0.983	df 1.000	pvalue 0.322
	Paenibacillus ~ Compost	cfi 1.000	tli 1.008	rfi 0.688
		nfi 0.938	srmr 0.055	AIC -8.746
	lavaan 0.6-11 ended normally after 31 iterations	rmsea 0	gfi 0.980	agfi 0.799
	Number of successful bootstrap draws 310			

The abbreviations in the table indicate the following: S_, soil; chisq, Chi-square: χ^2; df, degrees of freedom (DF); p-value, p values (Chi-square); cfi, comparative fix index (CFI); tli Tucker–Lewis index (TLI); nfi, (non) normed fit index; rfi, relative fit index (RFI); srmr, standardized root mean residuals (SRMR); AIC, Akaike information criterion; rmsea, root mean square error of approximation (RMSEA); gfi, goodness-of-fit index (GFI); and agfi, adjusted goodness-of-fit index (AGFI). Column No. 1 shows the best numerical structural equation model (Fig. 5c). Column No. 2 shows the inferior numerical structural equation model. Column No. 3 shows another inferior numerical structural equation model.

Table S4

A list of models targeted by causal mediation analysis for Fig. 5a and their statistical values.

Regression models	(I) L_Flavonoid ~ L_Aminoacid<br>(II) L_DPPH ~ L_Flavonoid<br>(III) L_Flavonoid + R_Carotenoid + L_DPPH ~ Compost

(I)	Estimate std.	Error	t value	Pr (> ltl)	
(Intercept)	-8.274	3.747	-2.208	0.05825	#
L_Aminoacid	22.876	6.428	3.559	0.00742	**

Residual standard error: 1.617 on 8 degrees of freedom
Multiple R-squared: 0.6128, Adjusted R-squared: 0.5645
F-statistic: 12.66 on 1 and 8 DF, p-value: 0.007416

(II)	Estimate std.	Error	t value	Pr (> ltl)	
(Intercept)	111.043	30.242	3.672	0.00629	**
L_Flavonoid	15.056	5.543	2.716	0.02641	*

Residual standard error: 0.004346 on 7 degrees of freedom
Multiple R-squared: 0.4798, Adjusted R-squared: 0.4147
F-statistic: 7.377 on 1 and 8 DF, p-value: 0.02641

(III)	Estimate std.	Error	t value	Pr (> ltl)	
(Intercept)	235.87	12.8	18.425	7.75E-08	***
Compost	-89.86	18.1	-4.963	0.0011	**

Residual standard error: 28.63 on 8 degrees of freedom
Multiple R-squared: 0.7549, Adjusted R-squared: 0.7242
F-statistic: 24.63 on 1 and 8 DF p-value: 0.001102

The non-parametric bootstrap confidence intervals and Quasi-Bayesian confidence intervals were not calculable. The coefficients and the related calculated data in the regression model are shown on the left. The values mediated among the regression models are shown on the right. The number in “Stimulations shows bootstrapping frequency. The abbreviations in the table indicate the following: T, treat; M, mediator; ACME, average causal mediation (indirect) effect; ADE, average direct effect; Total Effect, mediation (indirect) and direct effect; Prop.Mediated, proportion of mediated effect; #, p <0.1.

Table S5

A list of models targeted by causal mediation analysis for Fig. 5b and their statistical values.

Regression models	(I) L_Apigenin_7_O_neohesperidoside + R_Phenylalanine ~ R_2_Aminoadipate + Compost<br>(II) R_2_Aminoadipate ~ Compost<br>(III)L_Apigenin_7_O_neohesperidoside ~ L_Kaempferol + L_Quercetin_Glc

(I)	Estimate std.	Error	t value	Pr (> ltl)	
(Intercept)	6.956	3.086	2.254	0.0588	#
R_2_Aminoadipate	3.284	2.601	1.263	0.2472	
Compost	-3.551	1.359	-2.612	0.0348	*

Residual standard error: 1.603 on 7 degrees of freedom
Multiple R-squared: 0.7668, Adjusted R-squared: 0.7001
F-statistic: 11.51 on 2 and 7 DF, p-value: 0.006126

(III)	Estimate std.	Error	t value	Pr (> ltl)	
(Intercept)	-1.7507	0.3591	-4.875	1.80E-03	**
L_Kaempferol	3.2832	0.425	7.726	0.000114	***
L_Quercetin_Glc	-0.3031	0.8364	-0.362	0.727785	

Residual standard error: 0.2459 on 7 degrees of freedom
Multiple R-squared: 0.975, Adjusted R-squared: 0.9679
F-statistic:136.5 on 2 and 7 DF p-value: 2.469e-06

(II)	Estimate std.	Error	t value	Pr (> ltl)	
(Intercept)	1.15388	0.09748	11.838	2.38E-06	***
Compost	-0.34803	0.13785	-2.525	0.0355	*

Residual standard error: 0.218 on 8 degrees of freedom
Multiple R-squared: 0.4434, Adjusted R-squared: 0.3739
F-statistic: 6.374 on 1 and 8 DF, p-value: 0.03555

The non-parametric bootstrap confidence intervals and Quasi-Bayesian confidence intervals were not calculable. The coefficients and the related calculated data in the regression model are shown on the left. The values mediated among the regression models are shown on the right. The number in “Stimulations shows bootstrapping frequency. Th abbreviations in the table indicate the following: T, treat; M, mediator; ACME, average causal mediation (indirect) effect; ADE, average direct effect; Total Effect, mediation (indirect) and direct effect; Prop.Mediated, proportion of mediated effect; *, p<0.05; **, p<0.01; #, p <0.1.

Table S6

A list of models targeted by causal mediation analysis for Fig. 5c and their statistical values.

Regression models

(I) Compost ~ S_s_Methyl $_{\text{L}}$_cysteine + S_Nicotinamide + Paenibacillus
(II) Paenibacillus ~ Compost
(III) Compost + S_Nicotinamide ~ S_Aminoadipate
(IV) S_Nicotinamide ~ S_s_Methyl $_{\text{L}}$_cysteine

(I)	Estimate std.	Error	t value	Pr (> |t|)
(Intercept)	0.2428	1.2684	0.191	0.866
S_s_Methyl $_{\text{L}}$_cysteine	24.4635	23.1199	1.058	0.401
S_Nicotinamide	-5.7257	11.063	-0.518	0.656
Paenibacillus	2.4642	4.6093	0.535	0.646

Residual standard error: 0.4327 on 2 degrees of freedom
Multiple R-squared: 0.7503, Adjusted R-squared: 0.3758
F-statistic: 2.004 on 3 and 2 DF, p-value: 0.35

(II)	Estimate std.	Error	t value	Pr (> |t|)	
(Intercept)	0.1	0.03143	3.182	0.0335	*
Compost	0.11111	0.04444	2.5	0.0668	#

Residual standard error: 0.05443 on 4 degrees of freedom;
Multiple R-squared: 0.6098, Adjusted R-squared: 0.5122
F-statistic: 6.25 on 1 and 4 DF, p-value: 0.06677

(III)	Estimate std.	Error	t value	Pr (> |t|)
(Intercept)	1.3825	0.7001	1.975	0.12
S_Aminoadipate	-31.7255	25.9552	-1.222	0.289

Residual standard error: 0.506 on 4 degrees of freedom
Multiple R-squared: 0.2719, Adjusted R-squared: 0.08993
F-statistic: 1.494 on 1 and 4 DF, p-value: 0.2887

(III)	Estimate std.	Error	t value	Pr (> |t|)
(Intercept)	1.3825	0.7001	1.975	0.12
S_Aminoadipate	-31.7255	25.9552	-1.222	0.289

Residual standard error: 0.506 on 4 degrees of freedom
Multiple R-squared: 0.2719, Adjusted R-squared: 0.08993
F-statistic: 1.494 on 1 and 4 DF, p-value: 0.2887

Uncalculable on (I), (II),and (II) since the regression models did not have significant values (Nonparametric bootstrap Confidence Intervals and Quasi-Bayesian Confidence Intervals).

Nonparametric bootstrap Confidence Intervals
(treat: S_Nicotinamide; mediator: S_Methyl_L_cysteine)

(I) and (IV)	Estimate 95%	CI Lower 95%	CI Upper	p-value
ACME	1.31	-173.45	83.6	6.20E-01
ADE	0	0	0	1
Total Effect	1.31	-173.45	83.6	0.62
Prop. Mediated	1	1	1	NA

Sample Size Used: 6
Simulations: 1000

Quasi-Bayesian Confidence Intervals
(treat: S_Methyl_L_cysteine; mediator: S_Nicotinamide)

(I) and (IV)	Estimate 95%	CI Lower 95%	CI Upper	p-value
ACME	0	0	0	1.00E+00
ADE	-0.194	-2.909	2.55	0.92
Total Effect	-0.194	-2.909	2.55	0.92
Prop. Mediated	0	0	0	1

Sample Size Used: 6
Simulations: 1000

The non-parametric bootstrap confidence intervals and Quasi-Bayesian confidence intervals were not calculable. The coefficients and the related calculated data in the regression model are shown on the left. The values mediated among the regression models are shown on the right. The number in "Stimulations shows bootstrapping frequency. The abbreviations in the table indicate the following: T, treat; M, mediator; ACME, average causal mediation (indirect) effect; ADE, average direct effect; Total Effect, mediation (indirect) and direct effect; Prop.Mediated, proportion of mediated effect; *, p<0.05; #, p <0.1.

Table S7

Nif-related genes identified based on genomic data of *Paenibacillus macerans* HMSSN-036.

Contig No.	KEGG no.	Annotation	Information of annotation	Identity (%)
44	K04488	nitrogen fixation protein NifU and related proteins	>ref|WP_036624774.1| nitrogen fixation protein NifU [Paenibacillus macerans]	89
84	K01609	nitrogen-fixing NifU domain-containing protein	>ref|WP_036620067.1| nitrogen fixation protein NifU [Paenibacillus macerans]	95
87	K02585	nitrogen fixation protein NifB	>ref|WP_036628315.1| nitrogen fixation protein NifB [Paenibacillus macerans]	67
87	K02585	nitrogen fixation protein NifB	>gb|KFM95886.1| nitrogenase cofactor biosynthesis protein NifB [Paenibacillus macerans]	81
87	K02596	nitrogen fixation protein NifX	>ref|WP_036623755.1| nitrogen fixation protein NifX [Paenibacillus macerans]	79
87	K02596	nitrogen fixation protein NifX	>ref|WP_036623755.1| nitrogen fixation protein NifX [Paenibacillus macerans]	82
87	K02588	nitrogenase iron protein NifH [EC:1.18.6.1]	>ref|WP_036623760.1| nitrogenase reductase [Paenibacillus macerans]	94
87	K02588	nitrogenase iron protein NifH [EC:1.18.6.1]	>ref|WP_036623760.1| nitrogenase reductase [Paenibacillus macerans]	93
87	K02587	nitrogenase molybdenum-cofactor synthesis protein NifE	>ref|WP_036623757.1| nitrogenase iron-molybdenum cofactor biosynthesis protein NifE [Paenibacillus macerans]	85
87	K02587	nitrogenase molybdenum-cofactor synthesis protein NifE	>ref|WP_036623757.1| nitrogenase iron-molybdenum cofactor biosynthesis protein NifE [Paenibacillus macerans]	78
87	K02587	nitrogenase molybdenum-cofactor synthesis protein NifE	>ref|WP_036623757.1| nitrogenase iron-molybdenum cofactor biosynthesis protein NifE [Paenibacillus macerans]	86
87	K02592	nitrogenase molybdenum-iron protein NifN	>gb|KFM95816.1| nitrogenase molybdenum-iron cofactor biosynthesis protein NifN [Paenibacillus macerans]	79
87	K02592	nitrogenase molybdenum-iron protein NifN	>gb|KFM95816.1| nitrogenase molybdenum-iron cofactor biosynthesis protein NifN [Paenibacillus macerans]	84

Table S8

Nif-related genes identified based on genomic data of *Paenibacillus* sp. HMSSN-139.

<table>
<tr><th>Contig No.</th><th>KEGG no.</th><th>Annotation</th><th>Information of annotation</th><th>Identity (%)</th></tr>
<tr><td>6</td><td>-</td><td>nitrogen-fixing NifU domain-containing protein</td><td>>ref|WP_018754084.1| nitrogen-fixing protein NifU [Paenibacillus sanguinis]</td><td>93</td></tr>
<tr><td>13</td><td>K04488</td><td>nitrogen fixation protein NifU and related proteins</td><td>>ref|WP_036624774.1| nitrogen fixation protein NifU Paenibacillus macerans]</td><td>93</td></tr>
<tr><td>13</td><td>K04488</td><td>nitrogen fixation protein NifU and related proteins</td><td>>ref|WP_028538231.1| nitrogen fixation protein NifU [Paenibacillus sp. J14]</td><td>86</td></tr>
<tr><td>21</td><td>K02585</td><td>nitrogen fixation protein NifB</td><td>>gb|KFM95886.1| nitrogenase cofactor biosynthesis protein NifB [Paenibacillus macerans]</td><td>80</td></tr>
<tr><td>21</td><td>K02585</td><td>nitrogen fixation protein NifB</td><td>>ref|WP_036628315.1| nitrogen fixation protein NifB [Paenibacillus macerans]</td><td>71</td></tr>
<tr><td>21</td><td>K02596</td><td>nitrogen fixation protein NifX</td><td>>ref|WP_036623755.1| nitrogen fixation protein NifX [Paenibacillus macerans]</td><td>77</td></tr>
<tr><td>21</td><td>K02588</td><td>nitrogenase iron protein NifH [EC:1.18.6.1]</td><td>>ref|WP_036623760.1| nitrogenase reductase [Paenibacillus macerans]</td><td>93</td></tr>
<tr><td>21</td><td>K02587</td><td>nitrogenase molybdenum-cofactor synthesis protein NifE</td><td>>ref|WP_036623757.1| nitrogenase iron-molybdenum cofactor biosynthesis protein NifE [Paenibacillus macerans]</td><td>83</td></tr>
<tr><td>21</td><td>K02587</td><td>nitrogenase molybdenum-cofactor synthesis protein NifE</td><td>>ref|WP_036623757.1| nitrogenase iron-molybdenum cofactor biosynthesis protein NifE [Paenibacillus macerans]</td><td>86</td></tr>
<tr><td>21</td><td>K02592</td><td>nitrogenase molybdenum-iron protein NifN</td><td>>ref|WP_036623756.1| nitrogenase iron-molybdenum cofactor biosynthesis protein NifN [Paenibacillus macerans]</td><td>81</td></tr>
</table>

Table S9

Other functional gene candidates identified based on genomic data of *Paenibacillus macerans* HMSSN-036. The list shows some selected gene candidates associated with nitrogen cycle, phosphate solubilization, and siderophore reaction, amino acid synthesis, and auxin-related functions.

Contig No.	KEGG no.	Annotation	Information of annotation	Identity (%)
2	-	ferredoxin	>reflWP_036627952.1l ferredoxin [Paenibacillus macerans]	70
71	K05337	ferredoxin	>reflWP_036624247.1l ferredoxin [Paenibacillus macerans]	92
87	K02586	nitrogenase molybdenum-iron protein alpha chain [EC:1.18.6.1]	>reflWP_036623759.1l nitrogenase molybdenum-iron protein subunit alpha [Paenibacillus macerans]	91
87	K02591	nitrogenase molybdenum-iron protein beta chain [EC:1.18.6.1]	>reflWP_036623758.1l nitrogenase molybdenum-iron protein subunit beta [Paenibacillus macerans]	83
3	K14487	GH3 auxin-responsive promoter-binding protein	>reflWP_036618245.1l hypothetical protein [Paenibacillus macerans]	98
16	K07088	auxin efflux carrier	>reflWP_036625350.1l permease [Paenibacillus macerans]	95
103	-	auxin efflux carrier	>reflWP_036623550.1l aldo/keto reductase [Paenibacillus macerans]	97
113	-	auxin-induced protein PCNT115	>reflWP_010348235.1l aldo/keto reductase [Paenibacillus peoriae]	95
2	K01609	indole-3-glycerol phosphate synthase [EC:4.1.1.48]	>gblKFN09453.1l indole-3-glycerol phosphate synthase family protein [Paenibacillus macerans]	98.8
1	K01649	2-isopropylmalate synthase [EC:2.3.3.13]	>reflWP_036624250.1l transferase [Paenibacillus macerans]	99.8
61	K03293	amino acid transporter, AAT family	>reflWP_036625213.1l GABA permease (4-amino butyrate transport carrier) [Paenibacillus macerans]	98.2
189	K00823	4-aminobutyrate aminotransferase [EC:2.6.1.19]	>reflWP_036626357.1l aminotransferase class III [Paenibacillus macerans]	78
2	K03711	Fur family transcriptional regulator, ferric uptake regulator	>reflWP_036621828.1l Fur family transcriptional regulator [Paenibacillus macerans]	96
11	K03306	inorganic phosphate transporter, PiT family	>reflWP_036625809.1l inorganic phosphate transporter [Paenibacillus macerans]	93

Table S10

Other functional gene candidates identified based on genomic data of *Paenibacillus* sp. HMSSN-139.. The list shows some selected gene candidates asociated with nitrogen cycle, phosphate solubilization, and siderophore reaction, amino acid synthesis-related and auxin-related function.

Contig No.	KEGG no.	Annotation	Information of annotation	Identity (%)
5	K05337	ferredoxin	>ref|WP_009222802.1| MULTISPECIES: ferredoxin [Paenibacillus]	97
21	-	nitrogen fixation protein	>ref|WP_036623754.1| hypothetical protein [Paenibacillus macerans]	54
21	K02586	nitrogenase molybdenum-iron protein alpha chain [EC:1.18.6.1]	>ref|WP_036623759.1| nitrogenase molybdenum-iron protein subunit alpha [Paenibacillus macerans]	88
21	K02591	nitrogenase molybdenum-iron protein beta chain [EC:1.18.6.1]	>ref|WP_036623758.1| nitrogenase molybdenum-iron protein subunit beta [Paenibacillus macerans]	85
34	K07088	auxin efflux carrier	>ref|WP_009226239.1| permease [Paenibacillus sp. oral taxon 786]	95.8
15	K01609	indole-3-glycerol phosphate synthase [EC:4.1.1.48]	>gb|EES72334.1| indole-3-glycerol phosphate synthase [Paenibacillus sp. oral taxon 786 str. D14]	90.3
12	K03293	amino acid transporter, AAT family	>ref|WP_028539670.1| GABA permease (4-amino butyrate transport carrier) [Paenibacillus sp. J14]	90.6
55	K00823	4-aminobutyrate aminotransferase [EC:2.6.1.19]	>ref|WP_036644459.1| aminotransferase class III [Paenibacillus sp. oral taxon 786]	77
5	K01649	2-isopropylmalate synthase [EC:2.3.3.13]	>ref|WP_009222800.1| MULTISPECIES: 2-isopropylmalate synthase/homocitrate synthase [Paenibacillus]	95.1
2	K03711	Fur family transcriptional regulator, ferric uptake regulator	>ref|WP_036621828.1| Fur family transcriptional regulator [Paenibacillus macerans]	96
11	K03306	inorganic phosphate transporter, PiT family	>ref|WP_036625809.1| inorganic phosphate transporter [Paenibacillus macerans]	93

Table S11

Physicochemical indicators in the soil after cultivation of carrots.

Cat.	pH (H20)	EC mS/cm	CEC meq/100g	CaO mg/100g	MgO mg/100g	K_2O mg/100g	H_3PO_4 mg/100g	NO_3^- mg/100g	NH_4 mg/100g	Humus %	PAC	Zn mg/kg	Cu mg/kg	Fe mg/kg	Mn mg/kg	Total_N %	Total_C %
Control	6.32	0.27	15.7	438	72.1	29.5	199	0.2	1.9	2.3	1011	20.6	0.25	6.21	39.5	0.14	2.56
Compost	6.68	0.96	22.7	506	100	46.4	175	17.7	1.3	2.7	1528	22.1	0.21	4.33	35.6	0.15	2.93

The "PAC" in the table shows the data of Phosphate absorption coefficient.

Table S12

Statistical values of the structural equation models for the amino acids of the leaf and root.

Category	Model	Fit indices		
	R_Metformin + L_GABA ~ L_Aminoadipate + Compost	chisq 0.3	df 1.000	pvalue 0.584
	L_Aminoadipate ~ Compost	cfi 1.000	tli 1.145	rfi 0.949
No1		nfi 0.991	srmr 0.02	AIC -57.55
	lavaan 0.6-11 ended normally after 1 iterations	rmsea 0.000	gfi 0.999	agfi 0.993
	Number of successful bootstrap draws 978			
	R_Lysine + L_GABA ~ L_Aminoadipate + Compost	chisq 0.009	df 1.000	pvalue 0.924
	L_Aminoadipate ~ Compost	cfi 1.000	tli 1.257	rfi 0.998
No2		nfi 1.000	srmr 0.05	AIC 42.096
	avaan 0.6-11 ended normally after 1 iterations	rmsea 0.000	gfi 1.000	agfi 1.000
	Number of successful bootstrap draws 988			
	R_Lysine + L_GABA ~ R_L_Aminoadipate + Compost	chisq 0.131	df 1.000	pvalue 0.718
	R_L_Aminoadipate ~ Compost	cfi 1.000	tli 1.344	rfi 0.963
No3		nfi 0.994	srmr 0.017	AIC 20.070
	lavaan 0.6-11 ended normally after 2 iterations	rmsea 0	gfi 0.999	agfi 0.990
	Number of successful bootstrap draws 977			
	R_Arginine + L-GABA ~ L-Aminoadipate + Compost	chisq 0.201	df 1.000	pvalue 0.654
	R_L_2_Aminoadipate ~ Compost	cfi 1.000	tli 1.179	rfi 0.963
No4		nfi 0.994	srmr 0.018	AIC 46.055
	lavaan 0.6-11 ended normally after 2 iterations	rmsea 0	gfi 1.000	agfi 0.995
	Number of successful bootstrap draws 986			

Abbreviations in the table indicate the following: L_, leaf; R_, root; chisq, Chi-square: χ^2; df, degrees of freedom (DF); p-value, p values (Chi-square); cfi, comparative fix index (CFI); tli Tucker–Lewis index (TLI); nfi, (non) normed fit index; rfi, relative fit index (RFI); srmr, standardized root mean residuals (SRMR); AIC, Akaike information criterion; rmsea, root mean square error of approximation (RMSEA); gfi, goodness-of-fit index (GFI); and agfi, adjusted goodness-of-fit index (AGFI). Column No. 1 shows the best numerical structural equation model. Column No. 2 shows the inferior numerical structural equation model. Column No. 3 shows another inferior numerical structural equation model.
