# Supplementary material for: An agroecological structure model of compost—soil—plant interactions for sustainable organic farming
Source: ISME Commun. 2023 Mar 31;3:28. doi: 10.1038/s43705-023-00233-9 (PMC10066230; doi:10.1038/s43705-023-00233-9)
Supplement: Supplementary file 2 — Supplementary Methods [file 43705_2023_233_MOESM2_ESM.docx]

**Supplementary Methods**

**Title: An agroecological structure model of compost-soil-plant interactions for sustainable organic farming**

*Running head*: Sustainable Agriculture and Compost

Hirokuni Miyamoto*^1,2,3,4^, Katsumi Shigeta^5^, Wataru Suda^2^, Yasunori Ichihashi^6^, Naoto Nihei^7^, Makiko Matsuura^1,3^, Arisa Tsuboi^4^, Naoki Tominaga^5^, Masahiko Aono^5^, Muneo Sato^8^, Shunya Taguchi^9^, Teruno Nakaguma^1,3,4^, Naoko Tsuji^3^, Chitose Ishii^2,3^, Teruo Matsushita^3,4^, Chie Shindo^2^, Toshiaki Ito^10^, Tamotsu Kato^2^, Atsushi Kurotani^8,11^, Hideaki Shima^8^, Shigeharu Moriya^12^, Satoshi Wada^12^, Sankichi Horiuchi^13^, Takashi Satoh^14^, Kenichi Mori^1,3,4^, Takumi Nishiuchi^15^, Hisashi Miyamoto^3,16^, Hiroaki Kodama^1^, Masahira Hattori^2,17^, Hiroshi Ohno^2^, Jun Kikuchi*^8^, Masami Yokota Hirai*^8^

*Affiliations:*

*1. Graduate School of Horticulture, Chiba University, Matsudo, Chiba 271-8501, Japan*

*2. RIKEN Center for Integrative Medical Science, Yokohama, Kanagawa 230-0045, Japan*

*3. Sermas Co., Ltd., Ichikawa, Chiba 272-0033, Japan*

*4. Japan Eco-science (Nikkan Kagaku) Co., Ltd., Chiba, Chiba 260-0034, Japan*

*5. Takii Seed Co., Ltd., Konan, Shiga 520-3231, Japan*

*6. RIKEN BioResource Research Center, Tsukuba, Ibaraki 305-0074, Japan*

*7. Faculty of Food and Agricultural Sciences, Fukushima University, Fukushima, Fukushima 960-1296, Japan*

*8. RIKEN Center for Sustainable Resource Science, Yokohama, Kanagawa 230-0045, Japan*

*9. Center for frontier Medical Engineering, Chiba University, Chiba, Chiba 263-8522, Japan*

*10. Keiyo Gas Energy Solution Co., Ltd., Ichikawa, Chiba 272-0033, Japan*

*11.Research Center for Agricultural Information Technology, National Agriculture and Food Research Organization, Tsukuba, Ibaraki 305-0856, Japan*

*12. RIKEN, Center for Advanced Photonics, Wako, Saitama 351-0198 Japan*

*13. Division of Gastroenterology and Hepatology, The Jikei University School of Medicine, Kashiwa Hospital,* *Kashiwa, Chib*a 277-8567, Japan

*14. Division of Hematology, Kitasato University School of Allied Health Sciences, Sagamihara, Kanagawa 252-0329, Japan*

*15. Division of Integrated Omics research, Bioscience Core Facility, Research Center for Experimental Modeling of Human Disease, Kanazawa University, Kanazawa, Ishikawa, 920-8640, Japan*

*16. Miroku Co., Ltd., Kitsuki, Oita 873-0021, Japan*

*17. School of Advanced Science and Engineering, Waseda University, Tokyo169-8555, Japan*

* Hirokuni Miyamoto Ph.D. *Graduate School of Horticulture, Chiba University ; RIKEN Center for Integrative Medical Science; Sermas Co., Ltd.; Japan Eco-science Co. Ltd.*

**Email:**  hirokuni.miyamoto@riken.jp

* Jun Kikuchi, Ph.D. *RIKEN Center for Sustainable Resource Science*

**Email:**  [jun.kikuchi@riken.jp](mailto:jun.kikuchi@riken.jp)

* Masami Hirai, Ph.D. *RIKEN Center for Sustainable Resource Science*

**Email:** masami.hirai@riken.jp

***Cultivation and harvest survey***

The conceptual figure was illustrated in Fig. S2. The arable fields where the tests were carried out were well mixed by skilled collaborators and divided into two groups (0.6 m x 3 m = 1.8 m^2^ /group) at 30cm intervals in the specialized research farm (Takii Seeds Co., Ltd.) located in Konan city, Shiga Prefecture, Japan (N34°99′, E136°08′). To avoid differences in soil conditions between the two groups, the initial soil was carefully mixed by a skilled person, although the chemical values were not analyzed. A slow-release 11-11-7 fertilizer (Sumika Agrotech Co., Ltd.) (80 g/m²), PK 4-15-30-1 (Sumitomo Chemical Co., Ltd.) (40 g/m²), and bitter lime (Sumika Agrotech Co., Ltd.) (100 g/m²) were applied as the base fertilizer on 9 August 2016 before sowing. In the compost zone, thermophile-fermented compost powder (Miroku Co., Ltd., and Keiyo Gas Energy Solution Co., Ltd., Japan) [79] was applied at 15 g/m² simultaneously with the basal fertilizer. The chemical properties of the compost were as follows [80]: total C (carbon), 38.6 ± 1.9% as the mean ± SD; total N (nitrogen), 3.6 ± 0.5%; total P (phosphorus), 2.0 ± 0.5%; total K (potassium), 1.0 ± 0.1%; total Ca (calcium), 1.0 ± 0.1%; total Mg (magnesium), 0.7 ± 0.2%; and H_2_O (moisture), 16.0 ± 1.7%.

Carrot seeds (Takii Seeds Phytorich Series, Kyo Kurenai, Takii Seeds Co., Ltd.) were sown at 5 cm intervals along a row in the targeted cultivation area on 17 August 2016, and the rows were covered with non-woven fabric after sowing. The thinned seedling survey was carried out on 7 October 2016, and adjusted at 15cm intervals. The study was conducted under open field culture conditions, and moisture was basically supplied by rainfall. When the soil dried out and the crop began to wilt, watering was also applied, but not regularly and freely so that there were no differences between the two groups. The plants were harvested twice: the first harvest in 21 November 2016 and the second in 2 February 2017, and their stem and leaf weight, root weight, root diameter, and root length were measured.　Fresh weights were measured, and diameters were measured with vernier calipers for the largest root diameter. Just after the second harvest, the soil from two areas was randomly collected at approximately the midpoint between each individual plant with in each grouping test area. Soil was collected mainly at the 0-10 cm depth.

***RGB color image analysis***

The image analysis of carrots was performed with Adobe Photoshop software (<https://www.adobe.com>), ImageJ (https://imagej.nih.gov/ij/download.html), the library package “imager" in R software (<https://cran.r-project.org>), and the library packages “Spyder” (<https://www.python.ambitious-engineer.com/archives/>[2105](https://www.python.ambitious-engineer.com/archives/2105)) and “OpenCV” (<https://opencv.org>) in Python 3.8 (https://www.python.org). The background color of the photographs was changed to black using Photoshop. After first changing the background white (red = 255, green = 255, blue = 255) using Photoshop, the white background color was converted to black (red = 0, green = 0, blue =0). The png files were analyzed by the library package “imager", and RGB color conversion was then transformed. Based on calculation of the grayscale photos with RGB color indices, the histograms of red, green, and blue color were visualized. After identifying these differences in patterns, the following detailed analysis was conducted. The ImageJ was used to collect data with pixel values of at least 300 x 300 pixels per a carrot. The width of the sticker in the lower left corner of the photo was set as the standard (26 pixels). Additionally, after applying the Spyder and OpenCV using these data, the RGB color matrix values of the targeted portion (Fig. 1d) were arranged and extracted using the functions “imread” and “imwrite” for reading and writing images. The averages of the calculated values as the data for each carrot were used for RGB color image analysis.

***Taste survey***

The harvested carrot roots were randomly sampled and finely ground in a food processor, and the obtained juice was used for the survey. The samples were subjected to taste evaluation via a nondouble-blinded method according to the following indices: Sweet, sweetness; Rich taste, intensity of taste; Immaturity, green odor peculiar to carrots; Flavor, fragrance (scent) of the roots of the carrots. Four and eight evaluators took part in the evaluation in November and February, respectively. Pearson's chi-squared test was performed using the library “epitools” of the R software. Each value of X-squared, df, and p-value after the calculation is shown in the section of “Results”, respectively.

***Analysis of carotenoids***

In brief, carotenoid extraction was performed with the exception of some minor modifications according to the official protocol (<https://www.mext.go.jp/a_menu/syokuhinseibun/1368931.htm>). First, an accurately weighed edible sample (1.00 g) of each carrot was chopped into a few pieces, and the pooled samples were placed in a plastic tube (50 mL). Then, 1.0 mL of a 1% (w/v) sodium chloride (NaCl) solution and 15 mL of 3% (w/v) pyrogallol/EtOH were added to the tube, and the sample was well mixed using a Polytron homogenizer (Kinematica, Luzern, Switzerland) for approximately 1 min. Next, the sample tube was washed with 5 mL of 3% (w/v) pyrogallol/EtOH. After adding 2.0 mL of a 60% (w/v) potassium hydroxide (KOH) solution to the tube, the mixture was heated at 70°C for 30 min to achieve saponification. After cooling at room temperature, the mixture was divided into two plastic centrifuge tubes (50 mL). Then, 22.5 mL of a 1% (w/v) NaCl solution and 15 mL of hexane/ethyl acetate (9/1) were added to each tube, and carotenoids were extracted by shaking for 10 min. After centrifugation at 3500 rpm for 10 min, the upper layer was collected. Subsequently, 15 mL of hexane/ethyl acetate (9/1) was added to the tube containing the lower layer, and the extraction procedure (i.e., shaking and centrifugation) was performed again. The combined upper layer containing carotenoids was poured into a round-bottom flask, and the solvent was removed by evaporation using a rotary evaporator. Next, hexane/ethyl acetate (9/1) was added to dissolve the residue, and the mixture was transferred to a 10 ml measuring flask. The sample volume was then appropriately adjusted by adding hexane/ethyl acetate (9/1). The carrot extract solutions were stored at -80°C until HPLC analysis.

Lycopene, alpha-carotene, beta-carotene, and canthaxanthin standards for HPLC analysis originally produced by CaroteNature GmbH (Bern, Switzerland) were purchased from Wako Pure Chemical Ind. Ltd. (Tokyo, Japan). High-performance liquid chromatography (HPLC)-grade ethanol (EtOH), methanol (MeOH), and chloroform were provided by Wako Pure Chemical Ind. Ltd. (Tokyo, Japan). The other reagents and solvents used for carotenoid extraction and HPLC analysis were of analytical grade.

An aliquot (1.0 mL) of the extracted sample solution was filtered through a GL Chromatographic Disc 4P, 0.45 μm (GL Sciences Inc., Tokyo, Japan), to remove very small dust particles and insoluble matter, and the filtrate was dried using a centrifugal concentrator. The residue was resolved in 1000 μL of chloroform, and an aliquot (500 μL) was dried using a centrifugal concentrator. Thereafter, the residue was redissolved in 100 μL of chloroform, and an aliquot (90 μL) was mixed with 10 μL of canthaxanthin (0.1 mg/mL), whick was employed as the internal standard material for the present HPLC procedure to avoid any change in solvent volume during autosampler injection, and allow it to be corrected.

The HPLC equipment consisted of a HITACHI-HPLC system (Hitachi High-Tech Corporation, Tokyo, Japan) containing a pump, UV–VIS detector, column oven, and autosampler for sample injection. The employed HPLC procedure was similar to protocols described in the application sheets (LT028 and LT073) of GL Sciences Inc. (Tokyo, Japan). Analyses were performed in an Inertsil ODS-3 column (3 μm, 2.1 mm × 100 mm, GL Sciences Inc., Tokyo, Japan) with the mobile phase described below applied at a 0.2 mL/min flow rate. The mobile phase was a mixture of MeOH and EtOH (45/55). The column oven temperature, UV–VIS detector wavelength, and injection volume were 50°C, 455 nm, and 10 μL, respectively. Under these HPLC analysis conditions, the carotenoid peaks of the carrot samples were well separated. The amount of each carotenoid (i.e., lycopene, alpha-carotene, or beta-carotene) was calculated from the difference in the peak area ratios of an extract sample and a standard sample. The data were corrected based on the recovery of canthaxanthin (the internal standard).

***DPPH radical-scavenging assay***

As previously described [81], a carrot sample was homogenized with ethanol under ice-cold conditions, and the mixture was centrifuged at 2000 × g for 10 min. The upper ethanol layer was evaporated, and the volume of the extract solution was adjusted. Thereafter, 1.0 mL of distilled water, 1.0 mL of 50 mM Tris buffer solution (pH 7.4), and 1.0 mL of 0.1 mM DPPH solution in ethanol were added to the sample solution in the assay tube. The mixture was incubated at 37℃ for 20 min, and the absorbance at 517 nm was measured with an iMark microplate reader (Bio-Rad Co., Ltd., USA). Antioxidant activity was calculated from a calibration curve prepared using a set of standard colors obtained by mixing alpha-tocopherol in ethanol solution. Each value was expressed as the alpha-tocopherol equivalent per gram of sample (nmol/g carrot).

***Metabolome analysis***

As previously described [82], the plants were lyophilized and crushed with beads, and a sample of precisely 4 mg (lyophilized weight) was weighed. Subsequently, 1.0 mL of extraction solvent (0.1% formic acid in 80% methanol, including lidocaine (8.4 nmol/L) and 10-camphor sulfonic acid (210 nmol/L) as the internal standard) was added to the sample, and the mixture was centrifuged (1,000 rpm (9,100 g), 1 min). Then, 25 µL of the supernatant of the extract was transferred to a 96-well plate; 225 µL of extraction solvent was added; the samples were shaken, stirred (1,100 rpm, 6 minutes), and centrifuged (2,000 rpm, flashing); and 25 µL of the supernatant was transferred to a 96-well plate. Next, 250 µL of ultrapure water was added to the dry solid (10 minutes), shaken and redissolved (1,100 rpm, 6 minutes), and centrifuged (2,000 rpm, flashing). Then, 120 µL of the supernatant was transferred to a 384-well plate with a filter and centrifuged (2,000 rpm, 5 minutes). Next, plant (leaves and root) analysis was performed using a liquid chromatography (LC)-tandem quadrupole mass spectrometry (MS) system (LC: Acquity UPLC, MS: Xevo TQ-S, Waters). The sample introduction volume was 1 μL. The obtained raw data were selected according to certain conditions (peak area of plant sample > 3,000, peak area of extraction solvent > 1.000), and the data corrected based on the internal standards were used for analysis.

Metabolome analysis of soils was performed as previously described [83]. In brief, soil mixed with 10 ml of sterile water was filtered through a 5A filter (Advantec Co., Ltd.), and the filtrate was lyophilized and crushed with beads. An appropriate amount of extraction solvent was added according to the lyophilized weight. A sample of each mixture of 2.0 mg dry weight was prepared in a 1 mL volume. Finally, 1.0 mL of extraction solvent (0.1% formic acid in 80% methanol, including lidocaine (8.4 nmol/L) and 10-camphor sulfonic acid (210 nmol/L) as the internal standard) was added. The mixture was incubated for 2 min, centrifuged (1,000 rpm (9,100 g), 1 min), and stored frozen at -30°C. The supernatant (25 µL) of the extract was transferred to a 96-well plate; 100 μL of the extraction solvent was added; the mixture was shaken, stirred (1,100 rpm, 6 minutes), and centrifuged (2,000 rpm, flashing); and the supernatant (25 µL) was transferred to a 96-well plate. Next, 500 µL of ultrapure water was added to the dry solid (15 min), and the mixture was shaken, redissolved (1,100 rpm, 6 min), and centrifuged (2,000 rpm, flashing). Then, 120 µL of the supernatant was transferred to a 384-well plate with a filter and centrifuged (2,000 rpm, 2 minutes). Soil analysis was performed using an LC-MS system (LC: Nexera X2, MS: LCMS-8050, Shimadzu). The sample introduction volume was 1.5 µL. Peak checks were performed for all compounds. The raw data were corrected based on the internal standard, and the updated data were used for the analysis. These data were visualized by heatmapping and subjected to association analysis and SEM analysis, as described later.

***DNA extraction from soil***

The soil was collected after harvesting, and 5 g of each soil sample was added to a 15 mL tube. Five milliliters of sterile water was added, the soil was well suspended by vortexing, and the solution was filtered through filter paper (Advantech 5C) (retention particle size: 7μm) (Advantech Co., Ltd., USA) to remove large gravels and other solids that affect the DNA extraction procedure. The filtrate was collected in a new 15 mL tube and was freeze-dried. Using the freeze-dried samples, DNA was extracted with a QIAGEN Power Soil DNA Mini Kit (QIAGEN Co., Ltd., USA) according to the manufacturer's protocol.

***Meta sequence analysis of bacterial 16S rRNA gene sequences***

As previously reported [84], the V1-2 region of the bacterial 16S rRNA gene (27fmod-338r) was sequenced according to a previous report[84]. The amplified fragments were sequenced on an Illumina MiSeq system following the manufacturer’s instructions. The paired-end reads were merged using the fastq-join program based on overlapping sequences. Reads showing an average quality value of <25 and inexact matches to both universal primers were filtered out. Filter-passed reads were used for further analysis after trimming both primer sequences. The quality filter-passed reads of each sample were rearranged in descending order according to the corresponding quality values and then clustered into OTUs with a 97% pairwise identity cutoff using the UCLUST program, version 5.2.32 (<https://www.drive5.com>). The taxonomic assignment of each OTU was performed based on similarity searches against the Ribosomal Database Project (RDP) and the National Center for Biotechnology Information (NCBI) genome database using the GLSEARCH program. α-diversity indices of community richness (Chao1) and diversity (Shannon and Simpson) were calculated, and β-diversity indices were estimated via UniFrac analysis with weighted and unweighted principal coordinate analysis (PCoA). All 16S rRNA gene datasets were deposited in the GenBank Sequence Read Archive database as described in the Data availability. Phylogenetic trees were constructed on the basis of the mash distance using the neighbor-joining method as previously described [85, 86].

***Association analyses***

Association analysis is a technically established, elementary method of unsupervised learning that is also used for market research as a type of market basket analysis and is applied to achieve an understanding beyond the logic of numbers using relative numbers [87-90]. It is easy to apply when missing values are a characteristic of association analysis. Therefore, it may be a suitable approach when conditions are set such that the identity for classification is different for each categorized layer, for example, when metabolomic data and microbial population are analyzed. It can identify and classify associated components by subjecting them to conditions in which it is challenging to make horizontal comparisons. To predict the components associated with compost, an association analysis was performed as previously reported [87-90]. In brief, association analysis is an elementary way to infer an effect (“target”) from a cause (“source”). In this case, the “source” and “target” are represented as x and y, respectively, and the calculation factors for probability (P) are defined as follows:

support (x ⇒ y) = P(x ∩ y)

“support” is P(xy), joint probability (P) of co-occurrence

confidence (x ⇒ y) = P(x ∩ y)/P(x)

“confidence” is P(xy)/P(x), conditional probability (P) of occurrence of y after x has occurred.

lift (x ⇒ y) = P(x ∩ y)/P(x) P(y)

“lift” is P(xy)/P(x)P(y), measure of association/independence

A value of > 1 represents a positive association (if the value indicates independence, a value of < 1 represents a negative association).

Here, association rules were determined by using criterion values of support, confidence, and lift (“support = 0.2, confidence = 0.6, maxlen = 2” and “lift > 1.5”). The data combined with all information such as growth, color, taste, metabolites, and bacterial phyla and genera obtained with or without compost addition were used in the analysis. In this study, crop growth, color analysis, questionnaire survey, metabolome, and microbial analyses are performed for each categorized layer, which is suitable for association analysis as described above. To avoid the differences dependent upon the layers, omics data for the analysis were calculated based on the median value (M) within the data and sorted as 0 (< M) and 1 (> M). The growth, color, and taste data were evaluated a different times from the samples targeted for omics analysis. Additionally, the frequency with the number of samples is different. Therefore, when there was a significant difference in each analysis, the data were set as 0 (low level) and 1 (high level). Therefore, potentially associated components were explored via this analysis. The packages “arules” and "aruleViz" in R software were applied. The systemic network was rendered by Force Atlas with Noverlap in Gephi 0.9.2.

***Covariance Structure Analysis***

Covariance structure analysis/structural equation modeling (SEM) for confirmatory factor analysis (CFA) was conducted using the R software package “lavaan” [90-93]. The analysis codes refer to the website (<https://lavaan.ugent.be>). As a hypothesis for CFA, the groups selected via correlation, principal component, and association analyses were utilized as component candidates for the latent construct of metabolites and microbiota. The measured values of the metabolites and the relative abundance of the bacterial population were used for the statistical procedure. The treatments with and without compost amendment were set as 0 and 1, respectively. The model hypotheses were statistically estimated via maximum likelihood (ML) parameter estimation with bootstrapping (n = 1000) using the functions ‘ lavaan’ and ‘sem’. The “Number of successful bootstrap draws” in the table indicates the number of successful draws after calculation for standard error (1000 estimates as requested bootstrap draws). The model fit was assessed according to the chi-squared p value (p>0.05, nonsignificant), comparative fit index (CFI) (>0.9), Tucker–Lewis index (TLI) (>0.9), goodness-of-fit index (GFI) (>0.95), adjusted goodness-of-fit index (AGFI) (>0.90), root mean square error of approximation (RMSEA) (<0.05), and standardized root mean residual (SRMR) (< 0.08) as indices of good model fit [94]. The path diagrams of the good model were visualized using the package “semPlot” of R software [95]. The analyses of the estimated values were performed concerning ([https://www.pu-hiroshima.ac.jp/p/ttetsuji/R/[83]lavaan2sem.html](https://www.pu-hiroshima.ac.jp/p/ttetsuji/R/%5b83%5dlavaan2sem.html)), and the paths were visualized using the R software packages “DiagrammeR”, “htmlwidgets”, and “webshot” of R software.

***Isolation of nifH-positive thermostable bacteria***

For the isolation of nitrogen-fixing bacteria from compost, succinate liquid medium and succinate-BTB medium (pH 6.8) were prepared as follows: The following succinate liquid medium are amounts per 1000 ml. succinic acid (11.6 g), MgSO_4_・7H_2_0 (0.2 g), NaCl (0.1 g), CaCl_2_・2H_2_O (0.02 g), Na_2_MoO_4_ ・2H_2_O (40 mg/mL) (50 μL), MnSO_4_・H_2_O (20 mg/mL) (50 μL), biotin (0.2 L), Fe-EDTA MS (1 U) (9.6 mL) bromothymol blue (BTB) (0. 1 g/20 mL of EtOH) (1.5 mL), yeast extract (20 mg). Agar (15 g) was mixed in succinate liquid medium 1000ml. The mixture was sterilized and solidified after pH adjustment and used as succinate-BTB medium (pH 6.8).The following procedure for the preparation of soil sterile solutions was performed as follows: The soil in the research farm (Chiba University) located in Chiba city, Chiba Prefecture, Japan (N35°63′, E140°10′) was randomly collected and was gamma-sterilized with 25 kGly by cobalt 60 from Koga-isotope Co., Ltd., Shiga Prefecture, Japan (http://www.koga-isotope.co.jp). A few (about a teaspoon) of the compost powder (Miroku Co., Ltd., and Keiyo Gas Energy Solution Co., Ltd., Japan) [79] was incubated at 25°C for 6 days with or without 100 μg/ml of Kanamycin (Sigma Co., Ltd., USA) as follows: The γ-sterilized soil was diluted in succinate liquid medium (×100, ×1000, ×10,000 dilution), and 0.1 ml of the diluted soil-succinate liquid medium was inoculated into two Petri dishes of succinate BTB medium. The targeted bacteria were isolated from the colonies generated in the dishes. In brief, colonies were planted in 140 petri dishes and incubated. Then, one of the formed colonies was randomly taken from each Petri dish, and apparently different shapes were also isolated. As a whole, 192 colonies were collected from 140 Petri dishes. The presence of nifH was confirmed by direct PCR for all of these colonies. Direct PCR with a set of nifH primers was performed as previously described [96]. In addition, it was confirmed that the succinate BTB medium was blue in color. Two strains, *Paenibacillus macerans* HMSSN-036 and *Paenibacillus* sp. HMSSN-139 were selected. The former was isolated under Kanamycin-containing conditions, while the latter was isolated under non-kanamycin-containing conditions.

Based on these results, genomic analysis of the two strains was performed. The DNA samples from these isolates were extracted by a Power Microbial DNA Isolation Kit (MO Bio Laboratories Co., Ltd., USA) and purified by a QIAquik PCR Purification Kit (QIAGEN Co., Ltd., USA). Genome sequencing was performed as previously described [97] and deposited in the GenBank Sequence Read Archive database as described in the Data availability. The isolated strains were observed using scanning electron microscopy with some modifications based on an experimental protocol for the spore forming bacteria as previously reported [98, 99]. In brief, the preparation of the sample was implemented as follows: the samples of the bacterial solutions cultured on the petri dish were fixed in phosphate-buffered 2% glutaraldehyde and subsequently post-fixed in 2% osmium tetra-oxide for 2 hours in the ice bath. Then, the specimens were dehydrated in graded ethanol and dried by CO_2_ critical point drying. Finally, dried specimens were coated by osmium plasma ion coater and were submitted to be observed by Scanning electron microscopy (JSM-7500F, JEOL).

***Stable isotope analysis for nitrogen fixation***

As previously described, a cultivation test with Arabidopsis thaliana ecotypes Landsberg and Columbia was performed as previously described [80]. A commercial horticultural soil medium (Engei Baido; Kureha, Tokyo, Japan) (granules of diameter 2–3 mm) was used as the only soil for cultivation. The isotope test was performed according to the method described by Yano *et al*.[100]. The soil was mixed with compounds included in stable isotopes: 2 kg of soil, 116.6 mg of K^15^NO_3_ (Shoko Science Co., Ltd., Japan), and 150.15 mg of (^15^NH_4_)_2_SO_4_ (Shoko Science Co., Ltd., Japan). Approximately 25 to 30 seeds were sown in 180 g of the soil medium. The thermophile-fermented compost powder [79] was mixed well with the soil medium in the test group, and the soil was then watered. The pots were watered with distilled water *ad libitum*. The pots were transparently covered, incubated at 4°C for 24 h and vernalized under continuous illumination with a light intensity greater than 3000 lux at 23°C. Most of the seeds germinated after three days. The cover was removed four days after germination, and three seedlings were selected from each pot. These seedlings were watered every two days with approximately 100 mL of distilled water per pot and were grown for 21 days. The grown seedlings and the soils were freeze-dried and used to determine isotope contents. The isotopic composition of nitrogen and the ratio of ^15^N and ^14^N were determined by using a Flash 2000-DELTA^plus^ Advantage ConFlo III System (EA-IRMS; Thermo Fisher Scientific, USA; owned by Shoko Science Co., Ltd., Japan) according to the conventional protocol [90, 101-104].

***Biological assay for isolated Paenibacillus strains***

Biological assays of the production of auxin, the siderophore reaction, and phosphate solubilization were performed with some modifications as previously reported [105-107]. The auxin assay was performed using a procedure that was partly modified according to a previously described protocol [105] as follows. Sixty milliliters of a mixture of King B broth (tryptone, 1.2 g; K_2_HPO_4_, 0.069 g; MgSO_4_・7H_2_O, 0.09 g; glycerol, 0.9 g; L-tryptophan, 0.6 g) and Salkowski color developing solution (50 mL of 35% HClO_4_; 1 mL of 0.5 M FeCl_3_), and 50 μg/ml of auxin / 3-indole acetic acid (IAA) solution (Wako Co., Ltd.) was prepared. After 20 µL of the colony solutions of isolated *Paenibacillus* strains was added to 15 mL of King B broth, the broths were incubated at 80 rpm and 25°C for 48 h. The broths without and with the colony treatment were centrifuged at 10,000 rpm and 4°C for 10 min. Two milliliters of supernatant was mixed with 2 mL of the Salkowski color developing solution and 10 μL of phosphate and thereafter shielded from light and allowed to stand for 30 min at room temperature. Without changing these mixing ratios, additional experiments were performed with volumes of 1 ml, 1 ml, and 5 ul, respectively. After a calibration curve was drawn using IAA at 530 nm, the absorbance of the reaction solutions was measured. The calibration curve for the determination of IAA concentrations consisted of points at 0, 10, 20, 30, 40, 50, and 100 μg/ml. At the same time, the OD levels of the remainder of the solution (before centrifugation) after incubation in King B broth were aligned and compared by checking the approximate amount of bacteria at 600 nm.

The CAS assay for siderophore detection was performed with a procedure partly modified according to a previously described protocol [107] as follows. The 10x MM9 solution (KH_2_PO_4_, 3 g; NaCl, 5 g; NH_4_Cl, 10 g; D.W., 1 L), CAS solution (Mordant Blue 29, 605 mg; DW, 500 mL), cetyltrimethylammonium bromide (HDTMA) solution (HDTMA, 729 mg; D.W., 400 mL), FeCl_3_・HCl solution (1 mM FeCl_3_・6H_2_O, 10 mM HCl), and CAS assay agar (10 x MM9 solution, 100 mL; NaOH, 6 g; PIPES, 30.24 g; agar, 15 g; D.W., 750 mL) were prepared. Subsequently, 100 mL of FeCl_3_・HCl solution was added to 500 mL of CAS solution, and 400 mL of HDTMA solution was slowly mixed into the FeCl_3_・HCl and CAS solution. The mixture solution was used as the CAS-HDTMA solution. CAS assay agar was added to CAS-HDTMA solution and sterilized. At approximately 50°C, 10 mL of 20% glucose solution, 1 mL of 1 M MgCl_2_ solution, and 1 mL of 100 mM CaCl_2_ solution were mixed in an Erlenmeyer flask, and thereafter, 10 mL of CAS-HDTMA solution was slowly poured along the wall of the flask. The final solution was solidified on the plate for the CAS assay. To this plate, 20ul of the strain (>10^8^ cfu/ml) was added and incubated aerobically at 25°C. The plate was incubated for at least 14 days and judged when the colonies formed became positive in color (red or yellow) on the plate.

The NBRIP medium for the phosphate solubilization assay was prepared according to a previously described protocol [106]. NBRIP cultivation agar medium (glucose, 10 g; Ca_3_(PO_4_)_2_, 5 g; MgSO_4_·7H_2_O, 0.25 g; MgCl_2_·6H_2_O, 2.5 g; KCl, 0.2 g; (NH_4_)_2_SO_4_, 0.1 g; agar, 15 g) was prepared. The solution was sterilized and solidified on the plate for the phosphate solubilization assay. To this plate, 20ul of the strain (>10^8^ cfu/ml) was added and incubated aerobically at 25°C. The plate was incubated for at least 14 days and judged when the colonies formed became positive in color (transparent state) on the plate.

***Measurement of N_2_O from soil***

Fungi derived from banana stalks were cultured on potato dextrose agar (PDA) as previously described [79]. The fungi grown in two Petri dishes were dissolved in 15 mL of sterile water to prepare a fungal solution. Next, 10 mL of the fungal solution was diluted with 190 mL of sterile water. Subsequently, 1% PDA was added to 700 g of soil (Tanemaki Baido) (Takii Seed, Japan) to prepare PDA-containing soil in the container. Finally, 200 mL of the fungal solution was added to the PDA-containing soil. PDA-containing soil without any fungal solution was also included as a negative control. The soils without and with compost were adjusted as follows: 1) 4 mL of distilled water was added, and 2) 4 mL of compost solution was added. A compost solution was prepared by adding 40 mL of sterile water to 4 g of thermophile compost powder, after which the mixture was filtered into a 50-mL Falcon tube with a 100 µm cell strainer (Falcon Co., Ltd., Japan). Pots containing soil were inserted into a transparent sampling bag with two on-off valves (5 L: No. 1-6664-14) (As One Co., Ltd., Japan), and the pots and the bag (the valve side is on the bottom) were placed on a small table at an angle of approximately 30 degrees for seven days. After seven days, a 1-L aluminum bag (AAK-1) (GL Sciences Co., Ltd., Japan) was filled with nitrogen gas. The bag was then joined to a transparent sampling bag, and the aluminum bag was collected the next day (Condition I). After storage of these pots at 4°C for 1 month, the air inside was released, the bag was closed, and the stopper was closed; 2 h later, a new aluminum bag used for recovery was connected (Condition II). Measurement of the gas concentration in these aluminum bags was performed using a Picarro G5131-i analyzer (Picarro, Santa Clara, California, USA; <https://www.sanyo-si.com/products/maker/picarro/>, owned and supported by Sanyo Trading Co., Ltd., Japan) according to a previously reported protocol[108-110].

***Meta-sequence analysis of fungal communities in the soil***

The fungal communities in PDA-containing soil with the fungal solution (soil prepared for the experiment described in “Measurement of N_2_O from soil”) were determined based on the DNA sequence information as previously described [111]. In brief, DNA from　soils without (n=1) and with compost (n=1) selected randomly was extracted, and the fungal ITS1 region was amplified from each replicate with the ITS universal primer sets for fungal organisms established by GENEWIZ. The forward primer contained the sequence “GTGAATCATCGARTC” and the reverse primer contained the sequence “TCCTCCGCTTATTGAT”. DNA sequencing was also conducted by GENEWIZ. Inc., Japan. Taxonomic assignments and estimation of relative abundances from sequencing data were performed using the analysis pipeline of the QIIME software package (<https://docs.qiime2.org/>). All 16S rRNA gene datasets were deposited in the GenBank Sequence Read Archive database as described in the Data availability.

***Causal Mediation Analysis (CMA)***

Individual causal mediation relationships were calculated using the R software package "mediation" [90-93, 112] based on the tutorial website (<https://rpubs.com/Momen/485122>). In brief, the R software packages "mediation", "tidyverse", "knitr", and "caret" were used as previously described [90]. Each regression value in the SEMs was calculated by the 'lm' function. In the case of the significant values, the values of the relationships between components as mediators and outcomes were assessed using the 'mediate' function. As previously described, the estimated average causal mediation (indirect) effect (ACME), average direct effect (ADE), and proportion of total effect via mediation (Prop. Mediated) were calculated by quasi-Bayesian confidence intervals and nonparametric bootstrap intervals with 1000 stimulations ('sims=1000') as the numbers of calculations.

**BayesLiNGAM**

The BayesLiNGAM method [90, 93, 113], which is a Bayesian score-based approach, was applied for the causal structural inference among components in optimal SEMs as previously described. The BayesLiNGAM method was established by the "fastICA" package (<https://cran.r-project.org/web/packages/fastICA>) of R software. Based on the website information (<https://www.cs.helsinki.fi/group/neuroinf/lingam/bayeslingam/>), the percentage data calculated by BayesLiNGAM were visualized by the R package "igraph" as previously described ^90,93^.

**References**

79. Niisawa C, Oka S, Kodama H, Hirai M, Kumagai Y, Mori K, et al. Microbial analysis of a composted product of marine animal resources and isolation of bacteria antagonistic to a plant pathogen from the compost. J Gen Appl Microbiol. 2008;54:149-58.

80. Ishikawa K, Ohmori T, Miyamoto H, Ito T, Kumagai Y, Sonoda M, et al. Denitrification in soil amended with thermophile-fermented compost suppresses nitrate accumulation in plants. Appl Microbiol Biotechnol. 2013;97:1349-59.

81. Miyamoto H, Shimada E, Satoh T, Tanaka R, Oshima K, Suda W, et al. Thermophile-fermented compost as a possible scavenging feed additive to prevent peroxidation. J Biosci Bioeng. 2013;116:203-8.

82. Sawada Y, Tsukaya H, Li Y, Sato M, Kawade K, Hirai MY. A novel method for single-grain-based metabolic profiling of Arabidopsis seed. Metabolomics. 2017;13.

83. Uchida K, Sawada Y, Ochiai K, Sato M, Inaba J, Hirai MY. Identification of a Unique Type of Isoflavone O-Methyltransferase, GmIOMT1, Based on Multi-Omics Analysis of Soybean under Biotic Stress. Plant Cell Physiol. 2020;61:1974-85.

84. Kim SW, Suda W, Kim S, Oshima K, Fukuda S, Ohno H, et al. Robustness of gut microbiota of healthy adults in response to probiotic intervention revealed by high-throughput pyrosequencing. DNA Res. 2013;20:241-53.

85. Ondov BD, Treangen TJ, Melsted P, Mallonee AB, Bergman NH, Koren S, et al. Mash: fast genome and metagenome distance estimation using MinHash. Genome Biol. 2016;17:132.

86. Atarashi K, Suda W, Luo C, Kawaguchi T, Motoo I, Narushima S, et al. Ectopic colonization of oral bacteria in the intestine drives TH1 cell induction and inflammation. Science. 2017;358:359-65.

87. Shiokawa Y, Misawa T, Date Y, Kikuchi J. Application of Market Basket Analysis for the Visualization of Transaction Data Based on Human Lifestyle and Spectroscopic Measurements. Analytical Chemistry. 2016;88:2714-9.

88. Shiokawa Y, Date Y, Kikuchi J. Application of kernel principal component analysis and computational machine learning to exploration of metabolites strongly associated with diet. Sci Rep. 2018;8:3426.

89. Wei F, Sakata K, Asakura T, Date Y, Kikuchi J. Systemic Homeostasis in Metabolome, Ionome, and Microbiome of Wild Yellowfin Goby in Estuarine Ecosystem. Sci Rep. 2018;8:3478.

90. Miyamoto H, Asano F, Ishizawa K, Suda W, Miyamoto H, Tsuji N, et al. A potential network structure of symbiotic bacteria involved in carbon and nitrogen metabolism of wood-utilizing insect larvae. Sci Total Environ. 2022; <https://doi.org/10.1016/j.scitotenv.2022.155520:155520>.

91. Rosseel Y. lavaan: An R Package for Structural Equation. Journal of Statistical Software. 2012;48:1-36.

92. Rosseel Y, Jorgensen TD, Rockwood N, Oberski D, Byrnes J, Vanbrabant L, et al. Latent Variable Analysis. R package version06-9. 2021.

93. Miyamoto H, Kawachi N, Kurotani A, Moriya S, Suda W, Suzuki K, et al. Computational estimation of sediment symbiotic bacterial structures of seagrasses overgrowing downstream of onshore aquaculture. Environmental Research. in press.

94. Hooper D, Coughlan J, Mullen MR. Structural equation modelling: guidelines for determining model fit. Electron J Business Res Methods. 2008;6:53-60.

95. Epskamp S, Stuber S, Nak J, Veenman M, Jorgensen TD. Path Diagrams and Visual Analysis of Various SEM Packages' Output R package version112. 2019.

96. Mehta MP, Butterfield DA, Baross JA. Phylogenetic diversity of nitrogenase (nifH) genes in deep-sea and hydrothermal vent environments of the Juan de Fuca Ridge. Appl Environ Microbiol. 2003;69:960-70.

97. Ogata Y, Suda W, Ikeyama N, Hattori M, Ohkuma M, Sakamoto M. Complete Genome Sequence of Phascolarctobacterium faecium JCM 30894, a Succinate-Utilizing Bacterium Isolated from Human Feces. Microbiol Resour Announc. 2019;8.

98. Ichinose S, Tagami M, Muneta T, Mukohyama H, Sekiya I. Comparative sequential morphological analyses during in vitro chondrogenesis and osteogenesis of mesenchymal stem cells embedded in collagen gels. Med Mol Morphol. 2013;46:24-33.

99. Nishida A, Miyamoto H, Horiuchi S, Watanabe R, Morita H, Fukuda S, et al. Bacillus hisashii sp. nov., isolated from the caeca of gnotobiotic mice fed with thermophile-fermented compost. Int J Syst Evol Microbiol. 2015;65:3944-9.

100. Tanaka Y, Yano K. Nitrogen delivery to maize via mycorrhizal hyphae depends on the form of N supplied. Plant, Cell and Environment 2005;28:1247–54.

101. Satoh R, Suzuki Y. Carbon and Nitrogen stable analysis by EA IRMS. Res Org Geochem. 2010;26:21-9.

102. Simsek A, Bilsel M, Goren AC. 13C/12C pattern of honey from Turkey and determination of adulteration in commercially available honey samples using EA-IRMS. Food Chemistry. 2012;130:1115-21.

103. Nakashita R, Suzuki Y, Akamatsu F, Iizumi Y, Korenaga T, Chikaraishi Y. Stable carbon, nitrogen, and oxygen isotope analysis as a potential tool for verifying geographical origin of beef. Anal Chim Acta. 2008;617:148-52.

104. He F, Li M, He Y, Dong Z, Cao J, Dai Z, et al. Authentication of Processed Epimedii folium by EA-IRMS. J Anal Methods Chem. 2020;2020:8920380.

105. Malik DK, Sindhu SS. Production of indole acetic acid by Pseudomonas sp.: effect of coinoculation with Mesorhizobium sp. Cicer on nodulation and plant growth of chickpea (Cicer arietinum). Physiol Mol Biol Plants. 2011;17:25-32.

106. Nautiyal CS. An efficient microbiological growth medium for screening phosphate solubilizing microorganisms. FEMS Microbiology Letters. 1999;170:265-70.

107. Srimathi K, Suji HA. Siderophores Detection by using Blue Agar CAS Assay Methods. International Journal of Scientific Research in Biological Sciences. 2018;5:180-5.

108. Winther M, Balslev-Harder D, Christensen S, Priemé A, Elberling B, Crosson E, et al. Continuous measurements of nitrous oxide isotopomers during incubation experiments. Biogeosciences. 2018;15:767-80.

109. Lee A, Winther M, Priemé A, Blunier T, Christensen S. Hot spots of N2O emission move with the seasonally mobile oxic-anoxic interface in drained organic soils. Soil Biology and Biochemistry. 2017;115:178-86.

110. Arcand MM, Congreves KA. Elucidating microbial carbon utilization and nitrous oxide dynamics with 13C-substrates and N2O isotopomers in contrasting horticultural soils. Applied Soil Ecology. 2020;147.

111. Pauvert C, Buée M, Laval V, Edel-Hermann V, Fauchery L, Gautier A, et al. Bioinformatics matters: The accuracy of plant and soil fungal community data is highly dependent on the metabarcoding pipeline. Fungal Ecology. 2019;41:23-33.

112. Tingley D, Yamamoto T, Hirose K, Keele L, Imai K. mediation: R Package for Causal Mediation Analysis. Journal of Statistical Software. 2014;59:1-38.

113. Hoyer PO, Hyttinen A. Bayesian Discovery of Linear Acyclic Causal Models. arXivorg. 2009; <https://doi.org/https://arxiv.org/abs/1205.2641:arXiv:1205.2641>.
